# Crossing the Blood–Brain Barrier with Molecularly Imprinted Polymeric Nanocarriers: An Emerging Frontier in Brain Disease Therapy

**DOI:** 10.1002/advs.202517004

**Published:** 2025-11-29

**Authors:** Ranjit De, Shuliang Shi, Kyong‐Tai Kim

**Affiliations:** ^1^ School of Life Science Handong Global University 558 Handong‐ro Buk‐gu Pohang Gyeongbuk 37554 South Korea; ^2^ School of Life Science and Technology Herbin Institute of Technology Harbin 150001 China; ^3^ Generative Genomics Research Center Global Green Research & Development Center Handong Global University Pohang Gyeongbuk 37554 South Korea; ^4^ Department of Life Sciences Pohang University of Science and Technology (POSTECH) 77 Cheongam‐Ro Pohang Gyeongbuk 37673 South Korea

**Keywords:** blood‒brain barrier (BBB), molecular imprinting, molecular memory, polymer nanoparticles, synthetic antibody, targeted drug delivery

## Abstract

The ability to permeate the blood‒brain barrier (BBB) remains a major challenge in treating neurological disorders. Molecularly imprinted polymeric nanocarriers (nanoMIPs) are emerging as versatile platforms that integrate antibody‐mimetic recognition with exceptional stability, tunable physicochemical properties, and controlled drug release. This review summarizes recent advances in nanoMIP design, including template selection, polymerization strategies, and surface modifications, and explores their potential for targeted brain delivery. Particular emphasis is placed on surface engineering approaches, such as functionalization with apolipoprotein E (ApoE), transferrin, and angiopep‐2 ligands, which exploit receptor‐mediated transcytosis (RMT) to increase BBB permeation and drug accumulation in pathological brain regions. The therapeutic and diagnostic applications of nanoMIPs in neurodegeneration, brain tumors, and CNS infections are also highlighted. Finally, current limitations and future perspectives are discussed, including biocompatibility, large‐scale production, and regulatory considerations, positioning nanoMIPs as a next‐generation platform for overcoming BBB‐associated barriers, and advancing precision brain therapeutics.

## Graphical abstract

Molecularly imprinted polymeric nanocarriers (nanoMIPs) offer robust, antibody‐mimetic platforms to overcome the blood‐brain barrier. The article surveys nanoMIP design and ligand‐directed surface engineering that harness receptor‐mediated transcytosis, and highlights therapeutic and diagnostic applications in neurodegeneration, brain tumors and central nervous system infections, while addressing key challenges in biocompatibility, large‐scale manufacture and regulatory translation.

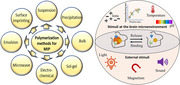

## 1 Introduction

**Figure 1:**
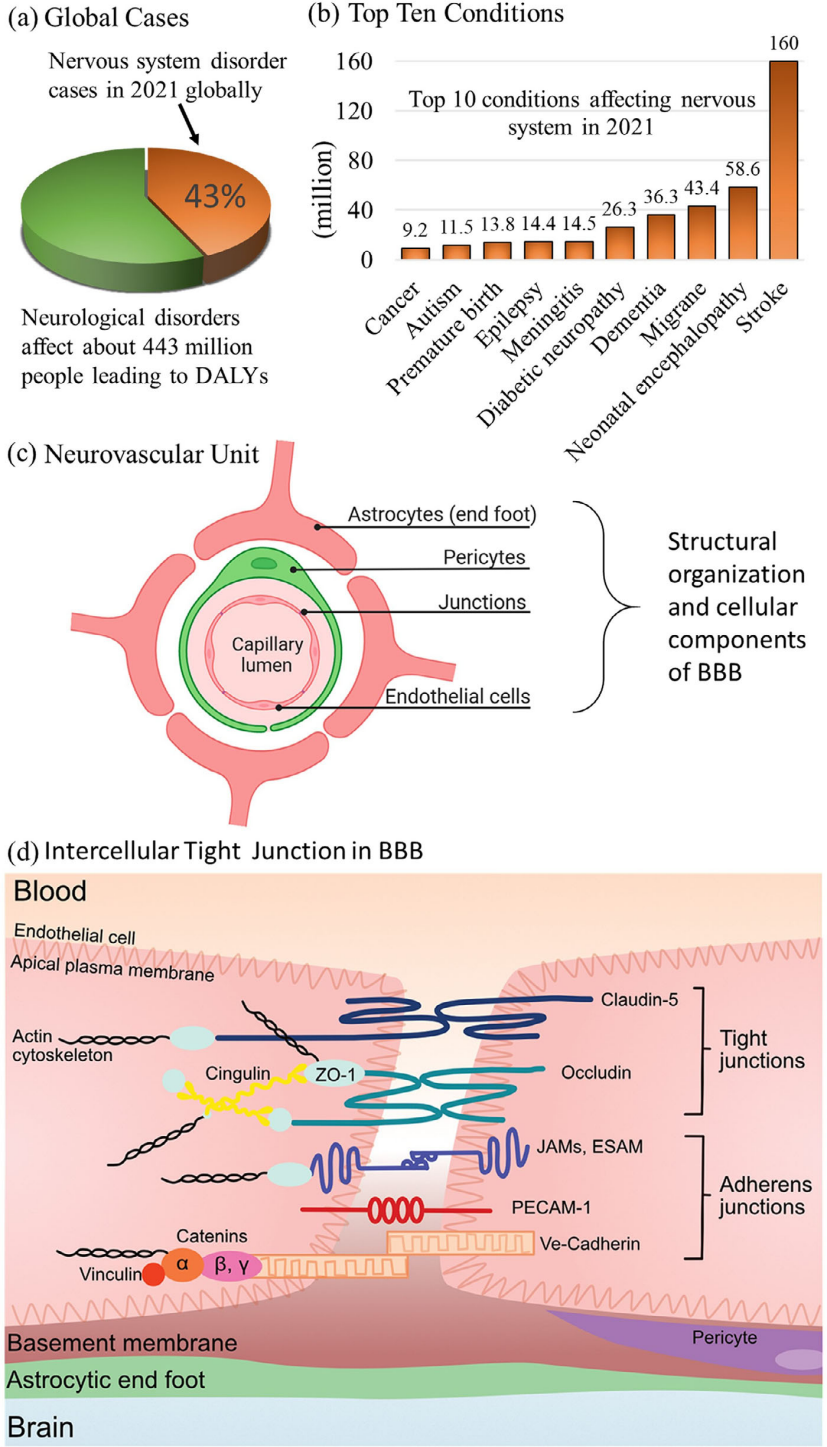
a) In 2021, ≈43% of the global population suffered from neurological conditions, which contributed to ≈443 million people with disability‐adjusted life‐years (DALYs). b) The top ten neurological conditions that accounted for the greatest DALYs resulted in a major global health crisis. The data presented in (a) and (b) were obtained from ref. [4]. c) A comprehensive schematic representation of the blood‒brain barrier (BBB) architecture. Reproduced with permission.^[^ ^7^ ^]^ Copyright 2023, Author(s). d) Schematic representation of the structural organization of the BBB and its intercellular tight junction complexes along with the adherens junction proteins. Reproduced with permission.^[^ ^10^ ^]^ Copyright 2022, Author(s).

Brain disorders represent an escalating global health challenge, contributing to nearly nine million deaths and disabilities annually across a diverse spectrum of neurological conditions.^[^ ^1^, ^2^ ^]^ These include neurodegenerative diseases such as Alzheimer's disease and Parkinson's disease, which progressively impair neuronal structure and function; malignant brain tumors such as glioblastoma and diffuse intrinsic pontine glioma (DIPG), characterized by rapid growth and invasiveness; and cerebrovascular disorders such as stroke, which disrupt cerebral blood flow and cause acute neurological deficits. Moreover, infectious and inflammatory conditions, including meningitis and autoimmune‐mediated demyelinating diseases such as multiple sclerosis, further contribute to the global burden of brain diseases.^[^ ^3^ ^]^ Each of these groups presents unique pathological features and therapeutic challenges, highlighting the complexity and multifaceted nature of brain disorders. Overall, they affect one in three individuals over their lifetime.^[^ ^1^ ^]^ According to a recent study published by the Global Burden of Disease 2021 Nervous System Disorders Collaborations, ≈3.4 billion people are affected by neurological conditions, which are equivalent to 43.1% of the global population, whereas the top ten neurological conditions that cause the most disability‐adjusted life‐years (DALYs) account for ≈361.7 million people (**Figure** **1**a,b).^[^ ^4^ ^]^ Despite decades of research, effective therapies for many neurological conditions remain palliative, addressing symptoms rather than halting disease progression.^[^ ^5^, ^6^ ^]^

### 1.1 The Restrictive Nature of the Blood–Brain Barrier (BBB)

A persistent challenge in the treatment of brain disorders is the blood‒brain barrier (BBB), a highly selective physiological interface that regulates the molecular exchange between the bloodstream and the central nervous system (CNS).^[^ ^7^, ^8^, ^9^ ^]^ Composed of tightly joined brain capillary endothelial cells supported by astrocytes and pericytes, the BBB effectively excludes the majority of small‐molecule drugs and nearly all macromolecular therapeutics from entering the brain parenchyma. This barrier selectively permits only a limited range of substances essential for maintaining CNS homeostasis, such as oxygen, glucose, amino acids, and some lipid‐soluble molecules. It typically restricts entry to compounds with molecular weights less than 500 Da.^[^ ^10^ ^]^ This impermeability significantly limits the efficacy of conventional treatments, necessitating the development of innovative approaches to achieve sufficient drug concentrations at diseased sites.^[^ ^11^ ^]^

The BBB is a highly specialized and dynamic interface that preserves the tightly regulated homeostasis essential for the CNS.^[^ ^12^, ^13^ ^]^ The endothelial cells in the BBB exhibit highly specialized structural and functional properties that distinguish them from peripheral endothelial cells (Figure 1c).^[^ ^7^ ^]^ In particular, they lack fenestrations, display extremely low rates of transcytotic vesicular transport or pinocytosis, and are enriched with a complex network of tight junction proteins. Among these proteins, claudins, occludins, and junctional adhesion molecules (JAMs) play central roles in maintaining barrier selectivity and integrity. Claudin‐5 is crucial for regulating size‐selective permeability, and its deficiency has been directly linked to increased paracellular leakage and compromised barrier function.^[^ ^9^, ^14^ ^]^ In addition to claudin‐5, other claudin isoforms, including claudin‐1, claudin‐3, and claudin‐12, also contribute to the fine‐tuning of BBB tight junction properties. Occludin and members of the membrane‐associated guanylate kinase (MAGUK) family, such as zonula occludens (ZO‐1, ZO‐2, and ZO‐3), perform essential scaffolding functions by linking transmembrane tight junction proteins to the actin cytoskeleton, thereby stabilizing intercellular junctions. Moreover, adherens junction proteins play complementary roles in the development, maturation, and structural organization of these intercellular connections. The key molecules in this category include vascular endothelial cadherins, catenins, platelet endothelial cell adhesion molecule‐1 (PECAM‐1), and various JAM isoforms (JAM‐A, JAM‐B, JAM‐C), as well as the endothelial cell‐selective adhesion molecule (ESAM) (Figure 1d).^[^ ^10^ ^]^ The high mitochondrial content within these cells supports the energy supply needed to operate the active transport systems required for maintaining ionic gradients and homeostasis.^[^ ^15^ ^]^

In addition to endothelial cells, pericytes and astrocytic endfeet are integral to BBB function. Pericytes contribute to angiogenesis, vessel stabilization, and tight junction formation, whereas astrocytes modulate endothelial behavior and regulate cerebral blood flow through neurovascular coupling.^[^ ^16^ ^]^ The restrictive nature of the BBB is further reinforced by efflux transporters such as P‐glycoprotein (P‐gp), breast cancer resistance protein (BCRP), and multidrug resistance‐associated proteins (MRPs), which actively expel xenobiotics.^[^ ^17^, ^18^, ^19^ ^]^ Carrier‐mediated and receptor‐mediated transport systems selectively import nutrients and peptides, while metabolic enzymes such as CYP450s degrade foreign molecules before they enter neural tissue.^[^ ^20^, ^21^ ^]^ Together, these features create a dynamic, multilayered barrier essential for CNS protection but challenging for therapeutic access (Figure 1d).

### 1.2 Limitations of Conventional Drug Delivery Strategies

Traditional drug delivery approaches face inherent structural and functional limitations imposed by the BBB.^[^ ^22^ ^]^ Dense tight junctions and a lack of fenestrations severely limit the passive diffusion of hydrophilic and large‐molecule drugs, whereas active efflux mechanisms (e.g., P‐gp, MRPs, and BCRP) further restrict intracerebral bioavailability.^[^ ^19^, ^23^ ^]^ Beyond these physical constraints, many agents suffer from poor metabolic stability and short plasma half‐lives, resulting in rapid systemic clearance and subtherapeutic brain concentrations.^[^ ^24^ ^]^ To compensate, higher systemic doses are often needed, which increases toxicity risks and reduces therapeutic precision. Moreover, the lack of site‐specific targeting in most conventional formulations leads to nonspecific drug distribution, compromising efficacy and increasing off‐target effects.^[^ ^25^, ^26^ ^]^

While invasive techniques such as intracerebral injections bypass the BBB, they pose risks of infection, hemorrhage, and tissue damage, making them unsuitable for widespread or chronic use.^[^ ^27^, ^28^, ^29^ ^]^ Noninvasive strategies, such as intranasal delivery, offer alternative routes of entry via the olfactory and trigeminal pathways but are constrained by limited dosing volumes and inconsistent transport efficiency.^[^ ^30^ ^]^ In a recent study by Francesco et al., ovalbumin delivery via intranasal administration in mice achieved ≈45% CNS permeation but remains difficult to generalize clinically.^[^ ^31^ ^]^ Moreover, the complex pathological features of brain diseases, including neuroinflammation, oxidative stress, protein misfolding, and heterogeneous tumor microenvironments, further hinder targeted intervention.^[^ ^32^, ^33^, ^34^ ^]^ These challenges highlight the urgent need for innovative, efficient, and minimally invasive delivery platforms capable of transporting therapeutics across the BBB to precise disease targets.^[^ ^35^ ^]^

### 1.3 Emergence of Nanotechnology and Molecular Imprinting in Biomedical Applications

**Figure 2:**
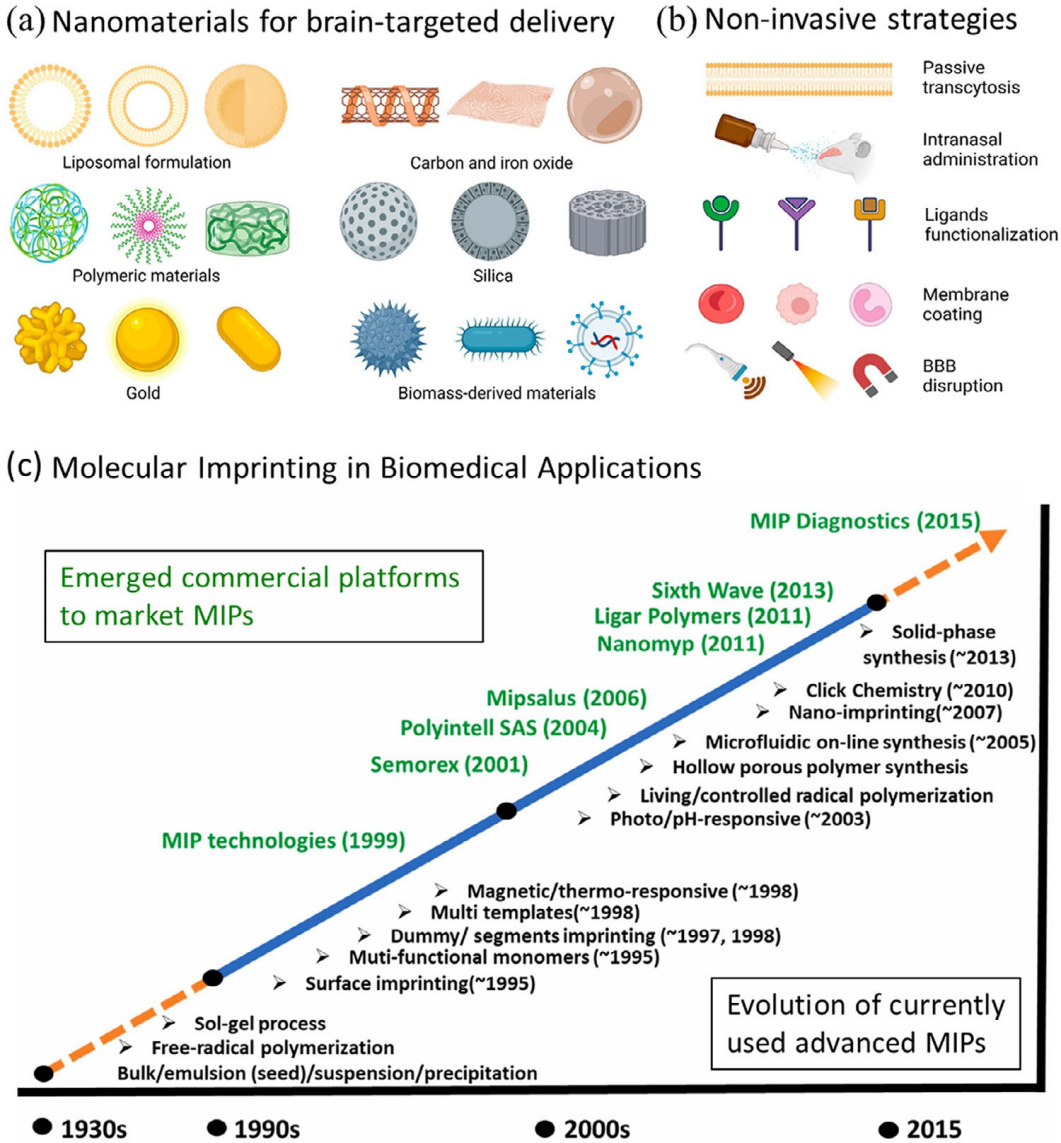
a) Schematic illustration of engineered materials of different shapes, sizes, morphologies, and materials for brain‐targeted drug delivery. Reproduced with permission.^[^ ^7^ ^]^ Copyright 2023, Author(s). b) Schematic diagram depicting various noninvasive techniques employed to facilitate therapeutic delivery across the BBB. Reproduced with permission.^[^ ^7^ ^]^ Copyright 2023, Author(s). c) Timeline illustrating the evolution of molecular imprinting processes and strategies leading to the development of advanced MIPs (below the line), along with the emergence of commercial platforms for diagnostics and other applications (above the line). Reproduced with permission.^[^ ^47^ ^]^ Copyright 2022, Elsevier.

Advances in nanotechnology and molecular imprinting are converging to redefine strategies for brain‐targeted therapeutics.^[^ ^36^, ^37^, ^38^, ^39^, ^40^, ^41^ ^]^ Nanotechnology, involving manipulation of materials at the 1–100 nm scale, enables precise engineering of particles compatible with biological systems.^[^ ^36^, ^42^, ^43^ ^]^ Advances such as scanning tunneling microscopy and the conceptualization of nanotechnology by Norio Taniguchi accelerated its translation from theory to biomedical innovation.^[^ ^44^ ^]^ Engineered nanomaterials are now widely explored for drug delivery, diagnostics, and biosensing, and their applications are illustrated in **Figure** **2** **a**.^[^ ^7^ ^]^ Furthermore, several noninvasive strategies used for drug delivery to the brain are illustrated in Figure 2b.^[^ ^7^ ^]^

Molecular imprinting technology, first conceptualized by Polyakov in the 1930s,^[^ ^45^ ^]^ has since evolved into a powerful method for creating synthetic receptors with template‐shaped recognition sites (Figure 2c).^[^ ^46^, ^47^ ^]^ Molecularly imprinted polymers (MIPs) mimic natural recognition systems, such as antigen‒antibody or enzyme‒substrate interactions, through the creation of template‒shaped cavities within polymer matrices.^[^ ^48^, ^49^ ^]^ Upon template removal, these sites exhibit precise molecular recognition, enabling applications in biosensing, separation, and targeted drug delivery.^[^ ^50^, ^51^ ^]^ The core principle involves creating a template of a target molecule, often a peptide, protein, or even an entire cell, within a polymer matrix. After template removal, a cavity complementary in size, shape, and chemical functionality is left behind, allowing for highly selective rebinding of the target molecule. This “lock and key” mechanism is the central aspect of the extensive utility of MIPs.^[^ ^52^, ^53^, ^54^ ^]^

The convergence of nanotechnology and molecular imprinting has yielded nanoMIPs, which synergistically increase specificity, stability, and functional versatility.^[^ ^55^ ^]^ Nanoparticles, owing to their high surface‐to‐volume ratios and tunable physicochemical properties, serve as ideal scaffolds for MIP integration.^[^ ^56^ ^]^ Core‐shell nanostructures based on magnetic materials, such as gold, silver, silica, or quantum dots, have been effectively coated with MIPs to improve their adsorption capacity, selectivity, and controlled release.^[^ ^57^, ^58^, ^59^ ^]^ For example, MIP‐coated silica nanoparticles for 17β‐estradiol achieved a fivefold increase in binding capacity and faster kinetics than nonimprinted analogs did, highlighting their promise for biosensing and analyte monitoring.^[^ ^50^ ^]^

Owing to their stability, reusability, and cost‐effectiveness, nanoMIPs represent a next‐generation platform for targeted drug delivery, diagnostics, and theranostics.^[^ ^42^ ^]^ Their ability to function as synthetic antibodies with high precision and adaptability positions them to address unmet challenges in brain‐targeted therapy.^[^ ^60^ ^]^

Furthermore, beyond polymeric and lipid systems, peptide‐based nanomaterials merit explicit emphasis for neuroscience and broader biomedical applications, as they can combine biodegradability, intrinsic biocompatibility, and tunable self‐assembly into architectures that enable precise physicochemical control, multifunctional payloading, and antibacterial/therapeutic activity. Recent reviews have highlighted advances in peptide‐derived nanobiomaterials, covering size‐shaped control, surface chemistry, and responsive behavior, alongside safety and degradability profiles, and demonstrated the utility of these materials from antibacterial coatings to CNS‐relevant delivery platforms.^[^ ^61^, ^62^, ^63^ ^]^ Therefore, the incorporation of peptide‐based materials in nanoMIP design can further expand their applications and make nanoparticles highly promising.

This review provides a comprehensive analysis of the integration of nanotechnology and molecular imprinting for biomedical applications, focusing on emerging strategies that employ MIPs as nanocarriers for drug delivery and diagnostics. Special attention has been given to protein‐ and peptide‐based MIPs and their relevance in addressing critical global health challenges, including the COVID‐19 pandemic and cancer. This review further aims to critically evaluate the methodologies, benefits, and limitations of various synthetic approaches for fabricating nanoMIPs. Finally, it identifies current challenges in clinical translation and outlines future perspectives for advancing these materials in biomedical contexts.

## 2 Structural Features of the BBB and Drug Delivery

The BBB is a complex and dynamic interface essential for maintaining cerebral homeostasis. Its specialized endothelial architecture, characterized by tight junctions and selective transport systems, strictly regulates molecular exchange between the bloodstream and the central nervous system (CNS).^[^ ^64^, ^65^ ^]^ Therefore, a comprehensive understanding of the anatomy and physiology of the BBB is essential for the rational design of drug delivery systems capable of achieving efficient and targeted transport of therapeutic agents to the brain.

### 2.1 Anatomy and Physiology of the BBB

This unique anatomical and physiological barrier was first described by Paul Ehrlich, who reported that certain dyes injected into the bloodstream stain peripheral organs but not the brain or spinal cord.^[^ ^66^ ^]^ Later, Edwin Goldmann further demonstrated this selectivity by showing that direct injection of trypan blue into the cerebrospinal fluid (CSF) stained CNS cells but not peripheral tissues.^[^ ^67^ ^]^ Therefore, the BBB ensures proper neuronal function and safeguards neural tissue from toxins, pathogens, and fluctuations in blood composition.

The BBB consists primarily of a specialized layer of brain microvascular endothelial cells that form the cerebral capillary walls. These cells are structurally distinct from their peripheral counterparts, exhibiting a flattened morphology, scarce vesicular transport, and a high mitochondrial content to sustain the energy demands of active transport (Figure 1c).^[^ ^7^, ^68^, ^69^ ^]^ Tight junctions are the primary structural component governing BBB selectivity. These proteins include claudins (especially claudin‐5), occludins, and JAMs, which are supported by cytoplasmic scaffolding proteins such as zonula occludens (ZO‐1, ZO‐2).^[^ ^10^, ^70^, ^71^ ^]^ These complexes seal the paracellular space, preventing the uncontrolled diffusion of polar molecules, whereas adherens junction proteins (e.g., VE‐cadherin, catenins, and PECAM‐1) complement tight junctions to maintain barrier stability (Figure 1d).^[^ ^10^ ^]^

The BBB operates within the context of the neurovascular unit (NVU), a functional assembly that includes pericytes, astrocytic endfeet, and neurons. Pericytes, which are embedded in the basement membrane, regulate angiogenesis, vessel maturation, and tight junction induction, and their dysfunction is linked to diseases such as Alzheimer's disease and stroke.^[^ ^72^, ^73^ ^]^

Astrocytes, which constitute ≈99% of the abluminal capillary surface, provide metabolic, structural, and neurotrophic support, modulating BBB properties and regulating the ionic and neurotransmitter balance essential for synaptic transmission.^[^ ^74^ ^]^ Their endfeet interact extensively with microvascular endothelial cells and pericytes. These astrocytes contribute to regulating the extracellular concentrations of neurotransmitters, metabolites, ions, pH, and water, which are crucial for synaptic transmission and neural function.^[^ ^75^, ^76^, ^77^ ^]^ For example, they participate in the spatial buffering of K^+^ ions, preventing potentially neurotoxic increases in extracellular potassium that can occur during neuronal activity.^[^ ^78^ ^]^ Thus, the complex interplay and signaling between endothelial cells, pericytes, and astrocytes form a dynamic and functional unit that collectively regulates the BBB's stringent permeability, enabling selective transport while preventing harmful substances from entering the brain.^[^ ^79^, ^80^ ^]^

### 2.2 Passive Diffusion versus Active Transport across the BBB

The BBB employs diverse mechanisms to regulate the passage of substances, primarily relying on passive diffusion for specific molecules and various active transport systems for others.^[^ ^81^, ^82^ ^]^ Understanding the distinctions between these transport modes is fundamental to developing effective brain‐targeting therapeutics. While it is often assumed that small molecules can cross the BBB, ≈98% of those, including most drugs, do not readily penetrate it.^[^ ^83^ ^]^

**Figure 3:**
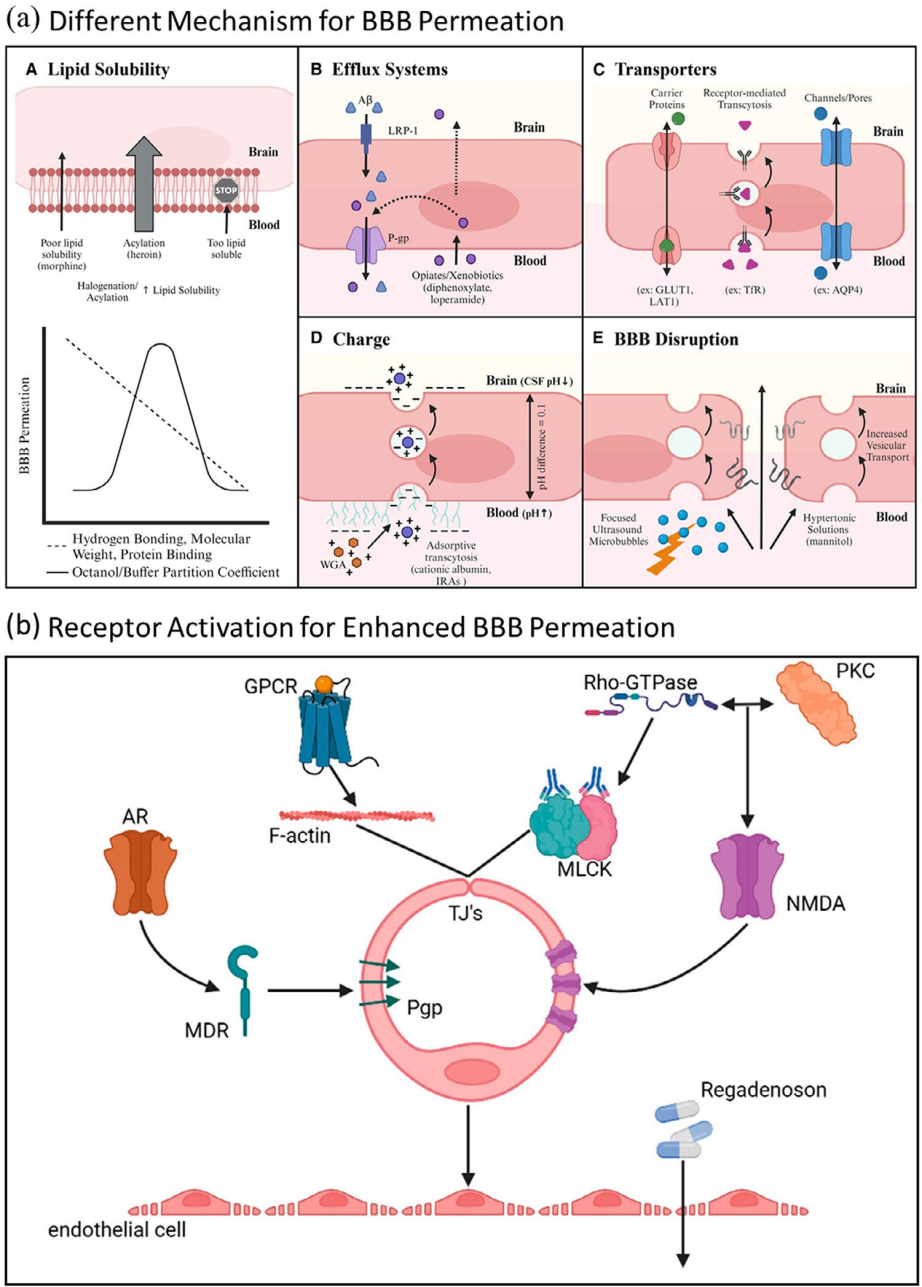
a) Schematic illustration of various mechanisms influencing BBB permeability and drug delivery: A) Passive diffusion of lipid‐soluble substrates and the effects of physicochemical properties; B) efflux transporter activity limiting CNS drug accumulation; C) receptor‐ and carrier‐mediated transport, including Trojan horse strategies; D) influence of substrate charge on adsorptive transcytosis and BBB penetration; and E) BBB disruption techniques such as hypertonic solutions and focused ultrasound with microbubbles. Reproduced with permission.^[^ ^85^ ^]^ Copyright 2024, Elsevier. b) Schematic representation of various approaches to increase BBB permeability, including activation of AR and NMDA receptors, widening of junctional gaps, and drug‐based modulation via agents such as Regadenoson. Reproduced with permission.^[^ ^121^ ^]^ Copyright 2024, Author(s).

Passive diffusion, the simplest form of transport, allows substances to move across the BBB along their concentration gradient without the expenditure of metabolic energy. This mechanism is highly dependent on the physicochemical properties of the compound, specifically its lipophilicity, molecular weight, and number of hydrogen bonds (H‐bonds) it can form (**Figure** **3**a).^[^ ^70^, ^83^, ^84^, ^85^ ^]^ Generally, only small, lipid‐soluble molecules with a molecular weight less than 400 Daltons can passively diffuse through the BBB.^[^ ^86^, ^87^ ^]^ The ability of a substance to permeate membranes decreases significantly with each pair of H‐bonds added to its structure, impacting its lipid solubility.^[^ ^9^, ^88^, ^89^ ^]^ For example, studies investigating the BBB transport of steroid hormones and oligopeptides have confirmed this rule.^[^ ^70^ ^]^ Compounds with a high polar surface area (PSA) greater than 80 Å^2^ and a tendency to form more than six hydrogen bonds are generally restricted from entering the CNS via lipid‐mediated free diffusion in therapeutically relevant amounts.^[^ ^9^ ^]^ This is because a significant increase in free energy is required to move such drugs from an aqueous phase into the lipid bilayer of the cell membrane.

In contrast, active transport mechanisms are energy‐dependent processes that transport molecules across the BBB, often against their concentration gradients, or facilitate the highly selective uptake of essential nutrients and the efflux of waste products and xenobiotics.^[^ ^7^, ^90^ ^]^ The BBB possesses a diverse array of specific influx and efflux transporters that play crucial roles in brain homeostasis and drug disposition. These include carrier‐mediated transporters (CMTs) and receptor‐mediated transport (RMT) systems (Figure 3a).^[^ ^85^, ^91^ ^]^ Carrier‐mediated transport systems facilitate the transcellular movement of essential water‐soluble nutrients and metabolites that cannot readily cross by passive diffusion. For example, glucose transporter‐1 (GLUT‐1) facilitates glucose uptake into the brain.^[^ ^92^ ^]^ This process is saturable because the number of available carriers limits the transport rate. Similarly, the large neutral amino acid transporter type 1 (LAT1) transports various large neutral amino acids, including phenylalanine, and has also been exploited for drug delivery, such as L‐DOPA, in Parkinson's disease treatment.^[^ ^93^, ^94^, ^95^ ^]^ Studies have shown that LAT1‐mediated transport can be targeted for novel prodrug development; for example, L‐tryptophan can be used as a promoiety for creating high‐affinity LAT1‐specific prodrugs.^[^ ^96^ ^]^ Furthermore, Peura et al. reported that phenylalanine derivatives of valproic acid could be designed to utilize LAT1‐mediated transport for brain targeting.^[^ ^97^ ^]^

Most receptors on brain endothelial cells are G protein‐coupled receptors (GPCRs), whose activation triggers calcium influx, leading to junctional gap formation and increased BBB permeability (Figure 3a).^[^ ^85^ ^]^ GPCR activation reorganizes F‐actin and disrupts tight junctions, facilitating peripheral drug entry into the brain. This process involves Rho‐GTPase and myosin‐light chain kinase (MLCK) signaling. Adenosine receptor (AR) activation plays a key role by (a) enhancing permeability to large molecules (e.g., via Regadenoson), (b) suppressing P‐gp expression through ubiquitination, increasing the accumulation of drugs such as epirubicin, and (c) potentially blocking immune cell entry when AR or CD73 (adenosine‐generating enzyme) is inhibited. Additionally, N‐methyl‐D‐aspartate (NMDA) receptor activation in brain endothelial cells can disrupt barrier integrity, likely via PKC and Rho‐GTPase signaling, enhancing both paracellular and transcellular transport. However, the exact NMDA receptor subtypes and kinetics of these responses are still unclear.

Conversely, active efflux transporters, predominantly members of the ATP‐binding cassette (ABC) superfamily, actively pump various endogenous substances and xenobiotics, including many therapeutic drugs, out of the brain capillaries back into the bloodstream.^[^ ^98^ ^]^ P‐gp, MRPs, and BCRP are key efflux transporters highly expressed at the BBB.^[^ ^19^, ^99^, ^100^ ^]^ These transporters significantly limit the brain penetration of a large number of drugs, posing a major hurdle in neuropharmacology. For example, Tang et al. reported that the accumulation of sunitinib, a multitarget tyrosine kinase inhibitor, in the brain is noticeably restricted by both ABCB1 and ABCG2 efflux activities.^[^ ^101^ ^]^ The overexpression of these efflux transporters is also implicated in the drug resistance observed in neurological disorders such as epilepsy.^[^ ^96^ ^]^ Therefore, strategies aimed at bypassing or inhibiting these efflux transporters are crucial for improving drug delivery to the brain.

### 2.3 Opportunities and Challenges in Transcytosis Pathways

In addition to passive diffusion limitations and active efflux by transporters, two critical vesicular transport processes, RMT and adsorption‐mediated transcytosis (AMT), present both opportunities and barriers for drug delivery to the CNS.^[^ ^102^, ^103^ ^]^ These physiological pathways, although primarily intended for the transport of essential molecules, can either facilitate or hinder the passage of drug delivery systems, depending on the systems’ design and their specific interactions with the BBB.^[^ ^104^, ^105^ ^]^

RMT is a highly specific, energy‐dependent process that utilizes endogenous receptors expressed on the luminal surface of brain endothelial cells to transport essential macromolecules from the blood into the brain.^[^ ^106^ ^]^ This pathway is initiated when a ligand, such as a natural protein or a therapeutic conjugate, binds to its specific receptor on the endothelial cell surface, triggering the internalization of the receptor‒ligand complex into clathrin‒coated vesicles or caveolae.^[^ ^107^ ^]^ These vesicles then traverse the endothelial cytoplasm and subsequently fuse with the abluminal (brain‐facing) membrane, releasing their cargo into the brain parenchyma.^[^ ^105^ ^]^ According to various studies, RMT systems involve receptors for insulin, transferrin (TfR), and low‐density lipoprotein (LDL).^[^ ^102^ ^]^ Choi et al. reported that monoclonal antibodies targeting TfRs can effectively transport conjugated drugs across the BBB, leveraging this endogenous pathway.^[^ ^108^ ^]^ However, a significant challenge with RMT is that many receptor‒ligand complexes are often sorted to lysosomes for degradation rather than completing transcytosis, leading to inefficient delivery.^[^ ^109^, ^110^, ^111^ ^]^ This intracellular trafficking dilemma means that while the drug‐carrying system may be internalized, it might not be effectively released into the brain parenchyma.^[^ ^112^ ^]^ Another issue is the high affinity of certain targeting antibodies, such as the 8D3 anti‐TfR antibody, which can lead to accumulation within endothelial cells rather than efficient transcytosis and may hinder their release into the brain.^[^ ^113^, ^114^, ^115^ ^]^

AMT, which is also known as nonspecific transcytosis, differs from RMT in its initiation mechanism, which relies primarily on electrostatic interactions.^[^ ^102^ ^]^ AMT is triggered by the binding of cationic molecules or nanoparticles to negatively charged domains on the luminal surface of brain ECs, such as the sialo‐glycoconjugates and heparan sulfate proteoglycans of the glycocalyx.^[^ ^102^, ^116^, ^117^ ^]^ This charge‒charge interaction induces nonspecific endocytosis, leading to the formation of vesicles that subsequently undergo transcellular transport. Cationic proteins and cell‐penetrating peptides (CPPs) are commonly employed in AMT‐based drug delivery strategies.^[^ ^118^ ^]^ These include cationized albumin, cell‐penetrating peptides derived from HIV‐Tat, and Syn‐B vectors, all of which have demonstrated the ability to cross the BBB via AMT.^[^ ^119^ ^]^ However, a notable limitation of AMT is its nonspecificity, which can lead to widespread tissue distribution and potential toxicity due to binding to anionic sites on other cell surfaces throughout the body. This can result in endothelial damage at high concentrations and nonspecific organ accumulation, compromising targeted delivery to the brain. Both RMT and AMT face challenges related to the fate of internalized cargo, including lysosomal degradation and inefficient release into the brain parenchyma, which can limit overall therapeutic efficacy.^[^ ^120^ ^]^

### 2.4 Modulating BBB Permeability via Endothelial Receptor Activation

Since brain endothelial cells play a crucial role in regulating BBB permeability through receptor‐mediated signaling pathways, activating their receptors could be a promising way to smuggle drugs across the barrier (Figure 3b).^[^ ^121^ ^]^ Among these receptors, G protein‐coupled receptors (GPCRs) are the most prominent, and their activation has been shown to induce calcium influx within endothelial cells. This triggers F‐actin reorganization and restructuring of junctional molecules, resulting in increased paracellular permeability and facilitating the transport of therapeutic molecules into the brain. For example, activation of the adenosine receptor (AR) promotes actin polymerization, which contributes to widening junctional gaps and enhances molecular passage. Furthermore, studies have demonstrated that these permeability changes are mediated through Rho‐GTPase activation and subsequent stimulation of myosin light chain kinase (MLCK), both of which disrupt tight junction integrity. Pharmacological interventions, such as the FDA‐approved drug Regadenoson, have also shown potential in transiently enhancing BBB permeability, enabling efficient delivery of large therapeutic molecules into the CNS. Conversely, the inhibition of ARs or CD73, an upstream enzyme responsible for adenosine generation, has been suggested as a strategy to reduce immune cell infiltration into the brain, highlighting the therapeutic versatility of these pathways.

Interestingly, adenosine receptor activation also appears to regulate efflux transporters such as P‐gp and other multidrug resistance (MDR) genes. By promoting the ubiquitination and redistribution of these proteins, AR activation suppresses their activity, thereby enhancing the accumulation of therapeutic drugs such as epirubicin within the CNS. In addition to GPCRs, NMDA receptors expressed on brain endothelial cells have been implicated in modulating BBB permeability. The activation of these receptors can modulate barrier integrity through protein kinase C (PKC) and Rho‐GTPase signaling pathways, potentially facilitating RMT and improving molecular delivery to the brain. However, the specific NMDA receptor subtypes involved and the kinetics of these responses remain to be fully elucidated.

Therefore, these findings suggest that targeting receptor‐mediated signaling pathways, particularly those involving GPCRs and NMDA receptors, offers promising avenues for enhancing BBB permeability and improving central nervous system drug delivery. Nonetheless, further studies are needed to better understand these approaches and ensure their safety and efficacy.

### 2.5 Current Approaches and Their Limitations

The current approaches to smuggling drugs across the BBB are either to temporarily disrupt the BBB, utilize endogenous transport pathways, or bypass the barrier entirely through alternative routes.^[^ ^122^ ^]^ While significant progress has been made, each method has inherent limitations that impact its clinical translatability, safety, and efficacy.

A direct but invasive approach involves temporary disruption of the BBB, typically achieved through osmotic agents such as mannitol or focused ultrasound (FUS) in combination with microbubbles.^[^ ^123^ ^]^ Osmotic disruption caused by the infusion of hyperosmolar mannitol solution causes shrinkage of brain endothelial cells and reversible opening of tight junctions, thereby increasing BBB permeability.^[^ ^124^ ^]^ While this method can achieve therapeutic drug concentrations in the brain, its major drawback is nontargeted, overall BBB disruption, potentially allowing harmful endogenous blood components and xenobiotics to enter the CNS, leading to neurotoxicity and impairing the neurobiological function of the BBB. Furthermore, the lack of patient‐friendliness and potential CNS injury are significant concerns. FUS, often combined with intravenously administered microbubbles, offers more localized and transient BBB opening.^[^ ^122^ ^]^ The mechanical oscillation of microbubbles under FUS pressure creates temporary disruptions in endothelial junctions, allowing drugs to penetrate. FUS has shown promise in clinical trials for delivering monoclonal antibodies for Alzheimer's and Parkinson's diseases. However, this method requires strict quality assurance to ensure consistency and safety, as evidenced by initial failures in acoustic coupling quality assurance in some glioma patients. Moreover, its long‐term safety and potential for cumulative negative effects from repeated exposures, particularly concerning vascular damage and neuroinflammation, are still under investigation.^[^ ^125^, ^126^ ^]^

Another set of strategies aims to leverage the natural transport systems of the brain, either by modifying drugs to mimic endogenous ligands or by encapsulating them within nanocarriers.^[^ ^127^ ^]^ Chemical modifications of drugs to increase lipophilicity have been explored to enhance passive diffusion. For example, the lipophilicity of the anticancer drug crizotinib, which has poor brain penetration ability, can be increased by conjugation with a fluoroethyl moiety, leading to increased BBB permeability.^[^ ^7^ ^]^ However, this approach can sometimes inhibit a drug's biological activity or lead to prolonged retention and side effects in nontarget peripheral organs due to increased biodistribution. Nanoparticle‐based delivery systems, including liposomes, polymeric nanoparticles, and solid lipid nanoparticles (SLNs), offer several advantages, such as high drug loading, improved biocompatibility, and prolonged circulation time.^[^ ^128^, ^129^, ^130^ ^]^ These nanocarriers can be engineered with surface modifications via specific ligands or antibodies to target receptors on the BBB (e.g., transferrin receptors and insulin receptors) to facilitate receptor‐mediated transcytosis. While promising, challenges remain in optimizing nanoparticle design for efficient transcytosis versus lysosomal degradation, ensuring targeted delivery to the brain parenchyma while minimizing accumulation in peripheral organs such as the liver or spleen.^[^ ^122^ ^]^ The “shuttle‐mediated” transport strategy, for example, aims to address the issue of the intracellular aggregation of nanoparticles within ECs, which can limit their effective passage.

Bypassing the BBB through alternative administration routes represents a noninvasive strategy. Intranasal (IN) administration is gaining attention because it allows drugs to reach the CNS directly from the nasal cavity via olfactory and trigeminal neuronal pathways, thereby circumventing the BBB and reducing systemic exposure and side effects. This route offers rapid delivery to the CNS, often within minutes.^[^ ^124^ ^]^ For example, Wu et al. reported that intranasally administered neurotrophins reduce neurodegeneration and improve cognitive function in animal models of Alzheimer's disease.^[^ ^7^ ^]^ However, IN delivery has limitations such as mucociliary clearance, drainage to the pharynx, variability in nasal cavity anatomy, and the limited volume that can be administered, which can affect the consistency and efficiency of drug delivery. Furthermore, health conditions such as allergies or cold nasal congestion can hinder its effectiveness. Despite these advances, a crucial predominant limitation for many of these approaches, particularly those involving nanoscale particles or complex drug modifications to increase BBB permeability, is their reproducibility and feasibility in large‐scale clinical applications, especially when moving from rodent models to human physiological structures. The current approaches for crossing the BBB along with their key limitations are presented in **Table** **1**.

**Table 1:** A table summarizing the mechanisms employed in current approaches for crossing the BBB along with their key limitations.

Approach	Mechanism	Advantages	Limitations
Receptor‐mediated transport (RMT)	Uses ligands or antibodies to bind specific BBB receptors (e.g., transferrin, insulin) for endocytosis	High specificity and efficiency	Receptor saturation, potential off‐target effects, limited cargo size
Carrier‐mediated transport (CMT)	Utilizes endogenous nutrient transporters (e.g., glucose, amino acids)	Exploits natural pathways	Competition with endogenous substrates, low transport capacity
Adsorptive‐mediated transcytosis (AMT)	Relies on electrostatic interactions between cationic ligands and negatively charged BBB membranes	Simple and versatile	Poor specificity, possible disruption of BBB integrity
Nanoparticle‐based delivery	Engineered nanoparticles (liposomes, polymeric, metallic) encapsulate drugs	Enhanced drug stability and controlled release	Risk of toxicity, immune response, limited BBB penetration
Cell‐penetrating peptides (CPPs)	Short peptides facilitate translocation across BBB cells	Can deliver diverse cargo types	Poor tissue specificity, potential cytotoxicity, rapid clearance
Focused ultrasound (FUS) and microbubbles	Temporarily opens the BBB via ultrasound‐induced cavitation	Noninvasive, localized BBB opening	Requires precise control, potential tissue damage, limited clinical adoption
Intranasal delivery	Direct transport via olfactory and trigeminal neural pathways	Bypasses BBB entirely, noninvasive	Low drug bioavailability, limited to small or lipophilic molecules
Exosome‐mediated delivery	Uses naturally derived vesicles for crossing the BBB	Biocompatible, low immunogenicity	Low loading efficiency, scalability issues, heterogeneity in exosome properties
Peptide/antibody conjugates	Drugs conjugated to peptides or antibodies for BBB targeting	Increased specificity	Complex synthesis, stability issues, limited clinical translation

## 3 Molecularly Imprinted Polymers (MIPs): Design Principles

**Figure 4:**
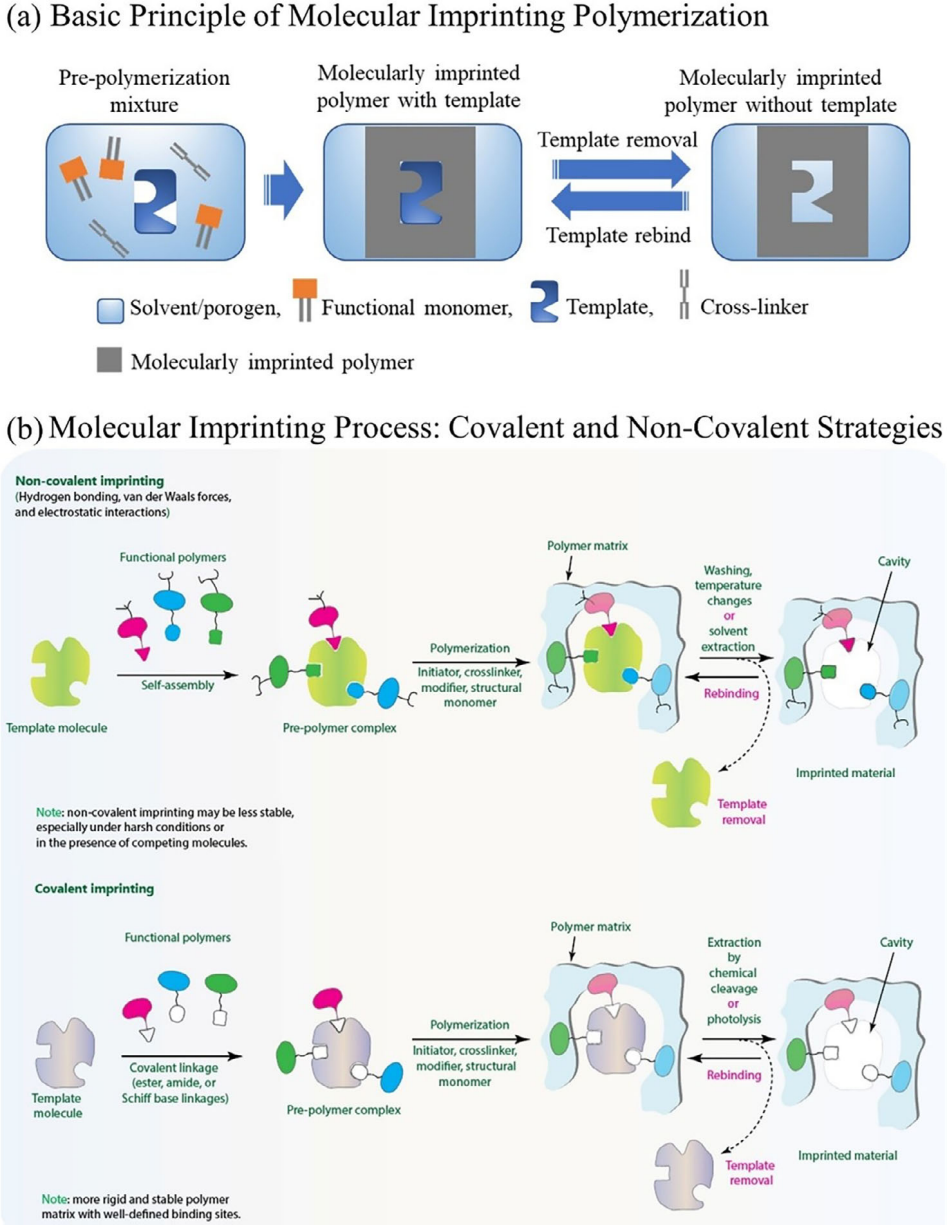
a) Schematic representation of the MIP preparation steps, including the formation of a prepolymerization mixture containing the template, functional monomer, and cross‐linker in a solvent, followed by polymerization to create a template‐complexed polymeric network and subsequent template removal and rebinding for cavity formation and evaluation. b) Overview of the molecular imprinting process employing covalent and noncovalent approaches, from template incorporation and polymerization to template removal, leading to the creation of highly specific recognition sites. Reproduced with permission.^[^ ^142^ ^]^ Copyright 2025, Elsevier.

MIPs represent a revolutionary class of “synthetic receptors” engineered to mimic the exquisite specificity and selectivity of natural biological recognition elements such as enzymes and antibodies.^[^ ^131^, ^132^, ^133^ ^]^ This technology allows for the creation of tailor‐made binding sites within a polymeric matrix designed to specifically recognize and bind target molecules. The fundamental principle of molecular imprinting is the formation of a precomplex between a template molecule and suitable functional monomers. In the presence of a cross‐linker and an initiator, a highly cross‐linked polymeric matrix is formed around this precomplex. Upon removal of the template molecule via appropriate desorption agents, a cavity is left behind within the polymer, which is complementary in size, shape, and chemical functionality to the original template (**Figure** **4**a).^[^ ^134^, ^135^ ^]^ This “molecular memory” enables the polymer to selectively rebind the target molecule, operating on the “lock and key” mechanism analogous to biological interactions.^[^ ^136^ ^]^

In addition to selectivity, MIPs offer several distinct advantages over their biological counterparts. They exhibit superior stability with several years of shelf life even under harsh environmental conditions, which include extreme pH values, high temperatures, and the presence of organic solvents, which often denature or degrade natural proteins and enzymes. This inherent robustness makes MIPs highly attractive for applications where biological receptors fail.^[^ ^137^ ^]^ Furthermore, MIPs are comparatively more cost‐effective to produce and highly reusable, contributing to their appeal for widespread implementation in various fields. The synthesis process can also be significantly less time‐consuming and labor‐intensive than the production of biological antibodies.

### 3.1 Molecular Imprinting Approaches: Covalent, Noncovalent, Semicovalent

There are several imprinting approaches, each offering unique benefits for specific applications. MIPs can be synthesized via noncovalent, covalent, or semicovalent strategies, each distinguished by how the template molecule associates with the functional monomer during polymerization and how it binds within the polymeric sites in the final product.^[^ ^138^, ^139^ ^]^ In these systems, molecular recognition can take place through interactions such as weak noncovalent hydrogen bonds, ion pairing, hydrophobic forces, and dipolar interactions (Figure 4b).^[^ ^140^, ^141^, ^142^ ^]^

#### 3.1.1 Covalent Imprinting

The covalent imprinting strategy involves the formation of reversible covalent bonds between the monomer and the template. Introduced by Wulff in 1972, this method creates a covalent linkage between the template and monomer that is subsequently cleaved during polymerization to remove the template from the MIP matrix.^[^ ^143^ ^]^ Rebinding of the target then relies on the reformation of this same covalent bond. A significant limitation of the covalent approach is the requirement for rapidly reversible covalent interactions, which restricts the range of suitable templates. Furthermore, the robust nature of these interactions can impede the achievement of thermodynamic equilibrium due to slow dissociation and binding kinetics. For example, a catechin sensor was developed by Senocak et al. using single‐walled carbon nanotubes (SWCNTs) covalently functionalized with a terminal ethynyl‐bearing subphthalocyanine (SubPc), creating a SWCNT‐SubPc hybrid material via a “click” reaction.^[^ ^144^ ^]^ This sensor demonstrated significantly increased differential pulse voltammetry responses to catechin compared with those of SWCNT‐modified glassy carbon electrodes (GCEs) and bare GCEs.

#### 3.1.2 Noncovalent Imprinting

The noncovalent imprinting approach is the most widely utilized approach because of its simplicity and versatility.^[^ ^145^ ^]^ This strategy, pioneered by the Mosbach group, relies on noncovalent interactions, such as hydrogen bonding, electrostatic forces, van der Waals forces, or hydrophobic interactions, to form an in situ complex between the template and functional monomer.^[^ ^146^, ^147^ ^]^ Synthesis typically involves mixing functional monomers, templates, cross‐linkers, and initiators in a suitable solvent. The key advantages of the noncovalent approach include its straightforward preparation, ease of template removal, rapid template binding to the MIPs, and broad applicability to various target molecules. However, careful selection of polymerization conditions is essential to ensure optimal formation of the labile template–monomer complex and minimize nonspecific binding sites. For example, the noncovalent molecular imprinting of sterols presents challenges because sterols generally possess only one hydrogen bond acceptor site and low polarity, which limits their ability to form strong association complexes with complementary functional monomers.^[^ ^148^ ^]^ Low‐temperature polymerization, often at 4 °C, is necessary for the noncovalent preparation of cholesterol‐imprinted polymers.^[^ ^149^ ^]^ Despite these challenges, noncovalently imprinted polymers are expected to exhibit faster rebinding kinetics.^[^ ^150^ ^]^

#### 3.1.3 Semicovalent Imprinting

The semicovalent imprinting method combines the benefits of both covalent and noncovalent approaches. In this strategy, the template is initially bound covalently during synthesis, and after its removal, rebinding occurs through noncovalent interactions. This approach aims to exploit the high affinity provided by covalent binding during synthesis while maintaining the mild operational conditions characteristic of noncovalent rebinding, offering a balanced approach. One mechanism for MIP synthesis via the semicovalent method involves free‐radical polymerization. For example, Qi et al. reported that MIPs for phenols were synthesized using a carbonyl group as a sacrificial spacer.^[^ ^138^ ^]^ The optimal MIP was prepared with 4‐chlorophenyl (4‐vinyl)phenyl carbonate as the template, ethylene glycol dimethacrylate (EGDMA) as the cross‐linker, 2,2‐azobisisobutyronitrile (AIBN) as the initiator, and chloroform as the porogen. Compared with its noncovalently imprinted counterpart, this semicovalently imprinted polymer exhibited superior selectivity for phenols and reduced peak broadening and tailing, suggesting its potential as a stationary phase for quantitative phenol determination. Another study comparing self‐assembly (noncovalent) and semicovalent approaches for benzylpiperazine MIPs revealed that semicovalent polymers had a stronger affinity and faster uptake.^[^ ^151^ ^]^

### 3.2 Common Techniques in MIP Design

**Figure 5:**
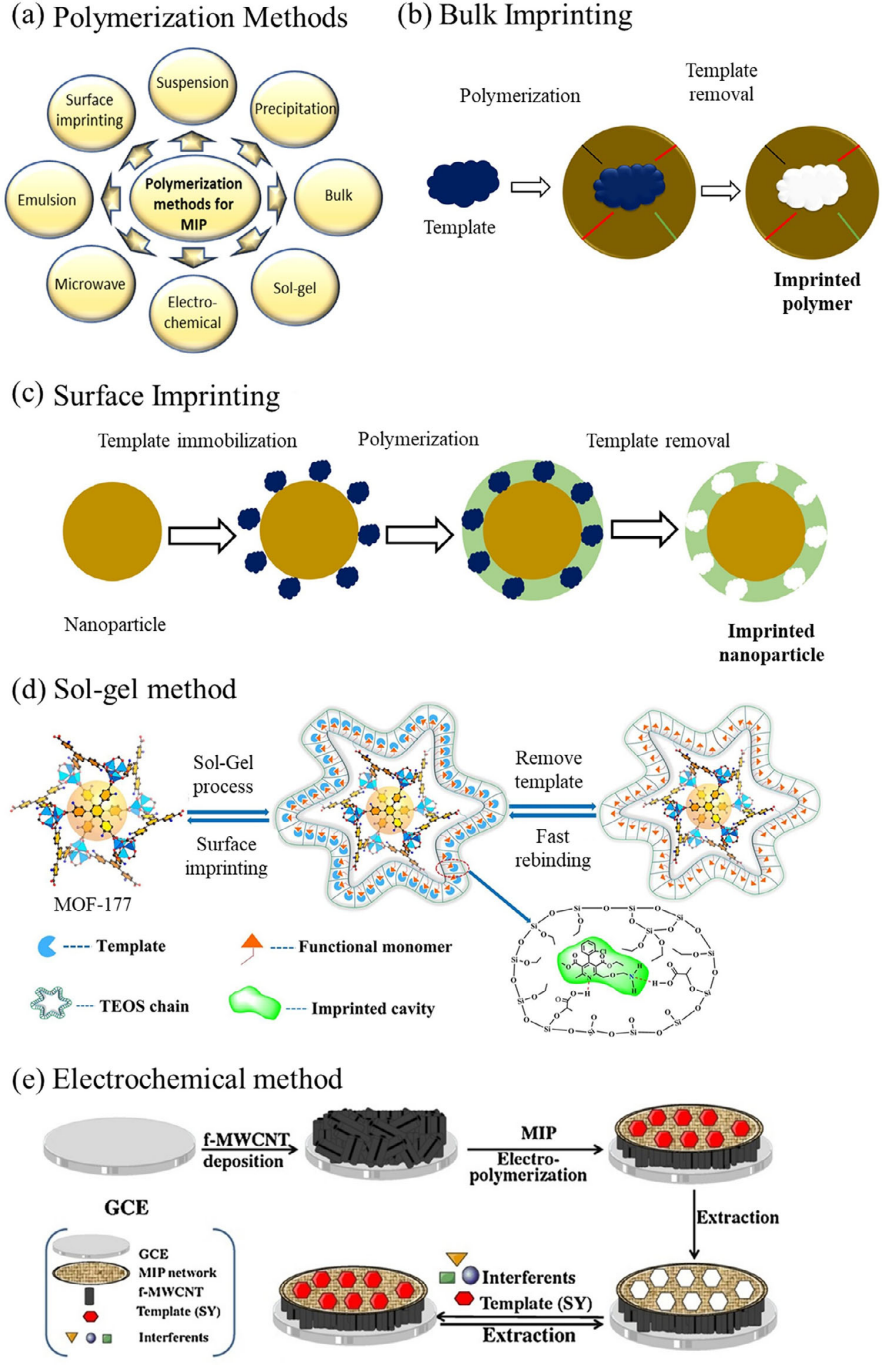
a) Schematic illustration of diverse polymerization strategies employed for the synthesis of molecularly imprinted polymers (MIPs). b) Overview of the synthetic steps involved in the preparation of MIPs via the bulk imprinting strategy. Reproduced with permission.^[^ ^153^ ^]^ Copyright 2021, Author(s). c) Surface imprinting approach for the fabrication of MIPs with highly accessible and specific recognition sites. Reproduced with permission.^[^ ^153^ ^]^ Copyright 2021, Author(s). d) Fabrication process of core‒shell sol‒gel hybrid MIPs constructed on a metal‒organic framework (MOF) scaffold. Reproduced with permission.^[^ ^195^ ^]^ Copyright 2021, Elsevier. e) Rapid electrochemical synthesis of MIPs anchored onto functionalized multiwalled carbon nanotubes (f‐MWCNTs). Reproduced with permission.^[^ ^210^ ^]^ Copyright 2017, Elsevier.

The availability of diverse polymerization techniques offers a wide range of approaches for conducting molecular imprinting polymerization, providing significant flexibility and advantages in MIP design (**Figure** **5**a). Among these methods, several widely adopted methods, such as bulk, precipitation, emulsion, suspension, electrochemical, and surface imprinting, are discussed below.^[^ ^152^ ^]^

#### 3.2.1 Bulk Imprinting

In bulk imprinting, the target analyte is directly incorporated into the polymer matrix during its formation.​ This conventional approach relies on the copolymerization of functional monomers and crosslinkers in the presence of template molecules (Figure 5b).^[^ ^153^ ^]^ The polymerization process is typically initiated through either thermal or photoinitiation, leading to the creation of a monolithic bulk polymer structure.^[^ ^134^ ^]^ While conceptually straightforward and relatively simple to implement, the bulk imprinting method presents certain practical limitations. A notable drawback is the substantial volume of reagents often required for the polymerization reaction, which can increase material costs, necessitate extensive purification processes, and generate considerable waste. Furthermore, the overall process can be time‐consuming because of the polymerization kinetics and subsequent steps, such as template removal.^[^ ^52^, ^154^ ^]^ For example, traditional bulk polymerization has been used to synthesize MIPs as porous monoliths.^[^ ^155^ ^]^ However, MIPs obtained by bulk polymerization often suffer from drawbacks such as an unequal distribution of recognition sites, an irregular morphology, incomplete template removal, and slow mass transfer.^[^ ^156^ ^]^ Despite these challenges, bulk imprinting has been successfully applied in various studies. For example, Liu et al. prepared three‐dimensionally ordered macroporous molecularly imprinted polymers by combining the colloidal crystal template method and the molecular imprinting technique.^[^ ^157^ ^]^ Compared with traditional bulk MIPs, this method results in a more regular macroporous structure, narrower macropore distribution, and greater surface area and porosity. Although protein imprinting via bulk techniques has been explored, challenges such as permanent entrapment, poor mass transfer, denaturation, and heterogeneity in binding pocket affinity have been identified as major drawbacks.^[^ ^158^ ^]^ Researchers have also synthesized shape‐selective pores in bulk silica by imprinting, demonstrating that shape selectivity can stem solely from the imprinted active site rather than the framework structure.^[^ ^159^, ^160^ ^]^ Despite its limitations, bulk imprinting remains a foundational technique in the development of molecularly imprinted polymers, and ongoing research continues to address its drawbacks to improve its efficiency and applicability.^[^ ^155^ ^]^

#### 3.2.2 Precipitation Polymerization

It is a widely used technique in MIP, where the template, functional monomers, crosslinker, and initiator are dissolved in a large volume of porogenic solvent.^[^ ^45^ ^]^ As polymerization continues, the growing polymer chains become insoluble and precipitate as uniform, spherical nanoparticles. This method offers several advantages, including the production of monodisperse particles with high surface areas and good binding site accessibility, without requiring stabilizers. The range of sizes of the particles can be wide, from the nanoscale to the microscale. Using this technique, Yang et al. reported the fabrication of nanoMIPs via a one‐pot strategy.^[^ ^161^ ^]^ In this study, propranolol‐imprinted nanoparticles were synthesized via various cross‐linkers in combination with methacrylic acid (MAA) as the functional monomer under reflux in acetonitrile. This synthetic approach yielded uniform propranolol‐imprinted nanoparticles within less than 3 hours, representing a substantial reduction compared with the conventional reaction time of 24 hours. Holdsworth et al. successfully prepared highly homogeneous nanoMIPs and evaluated their template incorporation, polymer composition, conversion, and binding performance.^[^ ^162^ ^]^ Although the use of a propranolol/methacrylic acid/EGDMA system has been demonstrated, the technique is broadly applicable to other precipitation‐based MIP formulations. Esfandyari‐Manesh et al. reported dipyridamole‐imprinted nanoMIPs prepared by precipitation polymerization, which exhibited higher affinity and slower release rates than nonimprinted polymers (NIPs).^[^ ^163^ ^]^ Compared with NIPs (17.1%), nanospheres (≈88 nm in the wet state; 50 nm dry; PDI 0.062) demonstrated significantly greater binding efficiency (62.7%) and superior imprinting capacity over irregularly shaped MIPs. In human serum, they bind ≈77% dipyridamole. Drug release studies revealed a controlled and sustained profile, with nearly complete release (99%) achieved within 17 days for nanoMIPs and 22 days for irregular particles, highlighting their promise for advanced drug delivery applications. Nevertheless, this method also has challenges such as high solvent consumption, lower polymer yield, potential template leakage, and limited compatibility with poorly soluble templates, which can restrict its scalability and environmental sustainability.^[^ ^164^ ^]^

#### 3.2.3 Emulsion Polymerization

Emulsion polymerization offers a robust and versatile pathway for synthesizing monodispersed nanoMIPs with strategically exposed binding sites.^[^ ^165^ ^]^ This technique is characterized by the dispersion of monomers in an immiscible liquid phase, typically water, aided by a surfactant. The process commonly involves the emulsification of a monomer within an aqueous surfactant solution. This approach is particularly advantageous for controlling the particle size and morphology, which are crucial for the performance of nanoMIPs. In conventional emulsion polymerization, surfactants play a critical role by promoting the emulsification of monomer droplets and solubilizing monomers within micelles, thereby controlling the number and size of the particles formed. For example, in 2000, Perez et al. pioneered the synthesis of nanoMIPs via emulsion polymerization, and a two‐stage miniemulsion polymerization method was subsequently used to create cholesterol‐imprinted (magnetic) nanoMIPs with diameters ranging from 50 to 210 nm.^[^ ^166^, ^167^ ^]^ This approach ensures an even distribution of binding sites on the surface of imprinted microspheres or nanoparticles, as also shown by Shen et al., which is vital for facilitating high reuse rates of the resulting MIPs.^[^ ^168^ ^]^ A notable variation is surfactant‐free emulsion polymerization, where polymerization occurs in the absence of emulsifiers.^[^ ^169^ ^]^ This technique is appealing for preparing polymer nanoparticles with narrow particle size distributions and well‐defined surface properties, eliminating impurities caused by residual emulsifiers and improving the water resistance of films. For example, the polymerization of styrene/oligoglycidol has been successfully carried out via surfactant‐free emulsion polymerization, showing its potential application for particle fabrication. Here, Pargen et al. successfully designed nanoparticles with sizes ranging from 100 nm to 600 nm.^[^ ^170^ ^]^ Similarly, polyacrylonitrile particles were synthesized by soapless emulsion polymerization of acrylonitrile in water, with the monomer and initiator concentrations affecting the conversion and microsphere diameter, which could be controlled between ≈140 and 500 nm.^[^ ^171^ ^]^ Recent advancements in surfactant‐free emulsion polymerization include photoinitiated processes, offering a sustainable alternative.^[^ ^169^ ^]^ Despite its advantages, the use of water and surfactants in emulsion polymerization can sometimes lead to undesirable precipitation. In some cases, apparent polymer precipitates are referred to as “precipitation polymerization,” where the polymer forms in the presence of a surfactant.^[^ ^165^ ^]^ Additionally, during emulsion polymerization, oligomers formed in the aqueous phase can reach a certain degree of polymerization and then precipitate to form primary particles, as shown by Krishnan et al.^[^ ^172^ ^]^ However, strategies exist to mitigate these issues, such as using reactive surfactants that become chemically bound to the polymer particles, reducing their desorption and minimizing film water absorption. Such modifications can improve latex characteristics and provide enhanced stability.

#### 3.2.4 Suspension Polymerization

Suspension polymerization is also frequently employed for the design and synthesis of nanoMIPs, yielding uniform and similarly sized microspheres from small colloidal droplets of the polymerization mixture suspended within a continuous liquid phase.^[^ ^173^ ^]^ The primary objective of this technique is to achieve a homogeneous distribution of spherical MIPs with effective binding properties. This method involves an organic phase comprising a monomer, cross‐linking agent, solvent, and initiator, along with an aqueous continuous phase that contains a surface‐active agent.^[^ ^174^ ^]^ When these phases are mixed, free‐radical polymerization begins and remains confined within the organic phase droplets, transforming the dispersed liquid droplets into spherical polymeric particles. Recently, Erdem et al. demonstrated that several key limitations in MIP synthesis, such as slow processing, low yield, and lack of in situ control, can be overcome via the use of a microreactor capable of continuously producing trillions of BSA‐imprinted nanoparticles within 5–30 min.^[^ ^134^ ^]^ Simulation studies revealed that the method yielded particles ranging from 52–106 nm with high precision, selectivity, and reusability. Notably, the system achieved substantial improvements in productivity, assay time, and reagent efficiency compared with conventional approaches. The presence of water in the continuous phase is particularly important, as it facilitates agitation and promotes heat transfer throughout the system. Suspension polymerization is generally advantageous because the reaction temperature is easily controllable, and the resulting particles exhibit high homogeneity and purity​.^[^ ^175^ ^]^ Alizadeh et al. used the same approach with some modifications to produce nanosized particles imprinted against the β‐adrenergic antagonist timolol.^[^ ^176^ ^]^ Here, they employed high‐speed mechanical and ultrasonic wave mixing to significantly reduce the droplet size, resulting in a modified carbon paste electrode that demonstrated strong and reversible target interactions. Recently, Sullivan et al. developed a microwave‐assisted suspension polymerization technique for the rapid production of imprinted nanoparticles.^[^ ^177^ ^]^ This method involves a toluene organic phase with MAA and steroid compounds as target templates and an aqueous continuous phase of polyvinyl alcohol (PVA). Polymerization was carried out at 110 °C in a microwave synthesizer after adding EGDMA and AIBN, and the synthesis time was reduced to only 45 minutes. This approach yielded nanoparticles with sizes between 120 and 143 nm. To produce water‐compatible nanoMIPs, the inverse suspension polymerization method has also been developed. This involves dispersing an aqueous phase (containing monomer and template molecules) in an organic phase (containing a surface‐active agent). Prasad and Pathak prepared multiwalled carbon nanotube‐functionalized pencil graphite electrodes (MWCNT‐PGE) modified with antitumor dacarbazine‐imprinted nanospheres (340 nm) via this technique.^[^ ^178^ ^]^ Polymerization was carried out at 70 °C in an aqueous phase of N‐acryloylamino butyric acid (ABA), dacarbazine (template), and 1,3‐diacryloylurea as a cross‐linking agent, with a cyclohexane continuous organic phase containing Span 80 as a stabilizer. However, a notable challenge with this method lies in precisely controlling the particle size distribution. This distribution is significantly influenced by several factors, including the type and concentration of the surface‐active agent, the quality of agitation, and physicochemical properties such as the density and interfacial tension between the phases. Furthermore, low productivity and the necessity for posttreatment to remove surface‐active agents, which can interfere with particle purity, are considered the main drawbacks.^[^ ^174^ ^]^ The possible intervention of template molecules in the continuous phase can reduce interactions between the monomer and the template, and the dispersing medium can severely affect the recognition ability of imprinted cavities. Nevertheless, ongoing research continues to seek solutions to these challenges.^[^ ^167^ ^]^

#### 3.2.5 Surface Imprinting

The surface imprinting technique (SIT) has emerged as an important strategy for designing nanoMIPs, effectively addressing the limitations associated with traditional bulk imprinting methods.^[^ ^179^, ^180^ ^]^ Unlike bulk imprinting, where the template is embedded within a monolithic polymer, SIT localizes recognition sites predominantly at or near the substrate surface (Figure 5c).^[^ ^153^, ^173^ ^]^ This strategy significantly enhances the performance of nanoMIPs by improving the accessibility of binding sites, enabling rapid binding kinetics, and ensuring complete template removal.

A notable advantage of SIT is its capacity to form composites with various nanomaterials, where nanomaterials can serve as sacrificial molds to achieve more precise control over the morphology and porosity of the imprinted polymer. This approach often involves the synthesis of the MIP shell on the surface of preformed nanoparticles, such as silica, iron oxide, or quantum dots.^[^ ^181^, ^182^, ^183^, ^184^ ^]^ For example, core‐shell nanoMIPs are frequently prepared by first forming a solid nanocore, followed by the grafting of a thin imprinted shell. This method allows for improved analyte transfer and offers better control over the thickness of the imprinted film than emulsion polymerization does. Recent studies have demonstrated the versatility of SIT. Bezdekova et al. reported the solid‐phase synthesis of nanoMIPs against pathogen surface monosaccharides, which is crucial for detecting specific biological targets.^[^ ^185^ ^]^ Additionally, a dispersive solid‐phase imprinting technique has been developed that employs magnetic particles instead of glass beads for template immobilization, resulting in high‐affinity, template‐free MIPs with higher yields.^[^ ^186^ ^]^ This technique involves immobilizing the template on in‐house synthesized magnetic particles instead of conventional glass beads, which results in high‐affinity, template‐free MIPs produced in relatively high yields. The solid‐phase synthesis of nanoMIPs represents an innovative method to prepare nanomaterials with tailor‐made molecular recognition sites, as demonstrated for rabbit IgG‐imprinted nanoMIPs.^[^ ^187^ ^]^ Using this technique, nanoMIPs have also been designed for the specific recognition of ochratoxin A (OTA).^[^ ^188^ ^]^ Furthermore, SIT has facilitated the development of soft molecularly imprinted nanoparticles, which exhibit hydrodynamic sizes of ≈100 nm, high surface‐to‐volume ratios, fast mass transfer kinetics, and limited dissipative dielectric properties.^[^ ^189^ ^]^ Investigations into vancomycin binding on nanoMIP surfaces via surface plasmon resonance‐based sensors highlight the precise control offered by SIT.^[^ ^190^ ^]^

SIT facilitates the creation of uniform, narrowly dispersed nanoMIPs with enhanced specific molecular‐recognition abilities, even in complex aqueous and biological samples.^[^ ^191^ ^]^ The integration of controlled living polymerization methods, such as reversible addition‒fragmentation chain transfer (RAFT) precipitation polymerization, with SIT has yielded highly monodisperse and efficient nanoMIPs.^[^ ^181^, ^192^ ^]^ Despite challenges in achieving optimal control over polymer film thickness and ensuring complete template extraction from dense polymer layers, SIT represents a significant step toward developing highly selective and robust synthetic receptors for diverse applications, advancing the design and application of nanoMIPs across fields such as diagnostics and biosensing.^[^ ^193^, ^194^ ^]^

#### 3.2.6 Sol‐gel Polymerization

Sol‐gel imprinting uses metal‒alkoxide precursors (e.g., tetraethyl orthosilicate and (3‐aminopropyl)triethoxysilane), which undergo acid‐ or base‐catalyzed hydrolysis and condensation to form an inorganic silica‐containing network around a template, yielding materials with highly controllable porosity, chemical and thermal robustness, and compatibility with aqueous media (Figure 5d).^[^ ^195^ ^]^ These properties can address the limitations of conventional organic MIPs, such as site collapse, poor porosity, and inadequate stability. Several studies have revealed that morphology/porosity control in silica‐containing matrices increases site accessibility and affinity, particularly in surface‐imprinted formats.^[^ ^196^ ^]^

Yu et al. have shown that sol‐gel MIPs can be used to detect melamine with high selectivity, demonstrating their feasibility for device integration.^[^ ^197^ ^]^

In another work, Santos et al. employed the sol‒gel method‐derived MIP for electrochemical caffeine sensing, where an imprinted siloxane on MWCNT‐modified electrodes provided sensitive, template‐specific responses, illustrating synergy between inorganic networks and nanocarbon transducers.^[^ ^198^ ^]^ Furthermore, Kia et al. prepared hybrid sol‒gel/organic MIPs as solid‒phase extraction sorbents for vitamin D_3_, which demonstrated high selectivity and efficient enrichment even in complex biological samples, thereby demonstrating the versatility and robustness of organic‒inorganic hybrid systems.^[^ ^199^ ^]^ Recent focused reviews further consolidate evidence that sol‒gel routes deliver MIPs with higher surface areas, improved mass transport, and enhanced durability across sample‐preparation and sensing platforms.^[^ ^200^ ^]^

#### 3.2.7 Electrochemical Polymerization

Electrochemical methods are widely employed for synthesizing conducting polymers because they offer precise control over material properties such as thickness, ion permeability, density, and porosity.^[^ ^201^, ^202^ ^]^ These methods allow for fine‐tuning of parameters such as the applied voltage, potential sweep rate, charge control, and duration and periods of applied potential pulses to manipulate the polymer thickness, ion permeability, density, and porosity.^[^ ^202^ ^]^ These properties can be tuned by adjusting parameters such as the applied voltage, potential sweep rate, charge control, and potential pulse duration, as well as by external treatments such as ultrasound or variations in ion and material concentrations. This versatile approach enables the preparation of intrinsically conducting polymers from diverse organic monomers via various techniques, including cyclic voltammetry, potentiostatic, and galvanostatic methods. A notable advantage is the direct deposition of polymer films onto metal surfaces, with the film thickness readily controlled through electrochemical parameters. For example, polypyrrole (PPy) can be synthesized electrochemically with precise control over its thickness and morphology.^[^ ^203^, ^204^, ^205^ ^]^ The process proceeds via the formation of a free radical cation that attacks a neutral monomer, followed by chain growth through reoxidation and proton loss. The overoxidation of conducting polymers is a crucial step in the formation of MIPs through electrochemical methods. This process facilitates the generation of oxidized radicals, which can increase the sensitivity and selectivity of the MIPs toward target molecules, as demonstrated by A. Yarman et al.^[^ ^206^ ^]^ Additionally, overoxidation aids in the removal of the template molecule, which is essential for creating specific binding sites and can also contribute to the regeneration of MIP‐based structures. Overoxidized polypyrrole membranes, for example, show a significant increase in C═O and COH functional groups and the presence of carboxylic groups, which influence the attachment of various species.^[^ ^207^ ^]^ Using this technique, Stephen et al. successfully designed nanoMIPs for protein sensing.^[^ ^208^ ^]^ This study presents a rapid (<2 h) one‐pot aqueous phase method for synthesizing nanoMIPs, which uses bovine hemoglobin as a model protein. The approach enabled production within 15 minutes, with the particle size controlled by the reaction termination time and fractionated via membrane filtration. The method is scalable, yielding 40 to 80 mg per batch of high‐affinity nanoMIPs, offering a fast and practical alternative to conventional production techniques. Thus, an emerging approach for preparing nanoMIPs involves solid‐phase polymerization, in which the template is covalently immobilized on a solid support, followed by rapid polymerization.^[^ ^209^ ^]^ The resulting nanoMIPs are then released, which results in high affinity and selectivity for the target molecule, serving as a robust alternative to antibodies. In another study by Arvand et al., MIPs were rapidly synthesized on carbon nanotubes via this electrochemical method, which was then successfully employed for the selective detection of sunset yellow molecules (**Figure** **5**e).^[^ ^210^ ^]^ Therefore, the sol‒gel method is promising for the rapid mass production of high‐affinity nanoMIPs. Since a detailed discussion of all the methods is beyond the scope of this work, a comprehensive analysis of several widely employed polymerization techniques suitable for nanoMIP synthesis is summarized in **Table** **2**.

**Table 2:** A comprehensive summary of the most widely employed techniques in molecular imprinting strategies, highlighting their respective advancements, limitations, and potential applications.

Polymerization method	Procedure	Advantages	Limitations	Ref.
Suspension polymerization	Monomer, cross‐linker, template, initiator mixed in organic phase, dispersed as droplets in aqueous or non‑aqueous dispersing medium under stirring.	Uniform spherical beads; strong mechanical integrity.	Requires emulsifiers/stabilizers; high solvent use; lower binding per surface area.	[211, 212]
Precipitation polymerization	Monomer, template, cross‐linker, initiator in porogenic solvent; as polymer forms it precipitates into uniform microspheres under dilute conditions.	Monodisperse particles; no stabilizers needed; easy purification; high selectivity.	Requires large solvent volumes; limited scalability; modest yields.	[213, 214]
Bulk Polymerization	Template, monomer, cross‐linker, initiator, porogen mixed and polymerized in one mass; resulting monolith is crushed and sieved into particles.	Simple and cost‐effective; high binding capacity; stable.	Grinding damages sites; irregular particle size; heterogeneous morphology.	[215]
Sol‐Gel Polymerization	Sol–gel precursors (e.g., TEOS), template, functional monomers undergo hydrolysis–condensation, forming a porous inorganic/organic matrix.	High thermal/chemical stability; gentle conditions; narrow pore sizes.	Difficult template removal; shrinkage/cracking on drying; poor for hydrophobic templates.	[216]
Electrochemical Polymerization	Functional monomer + template electropolymerized on conductive electrode via cyclic voltammetry or potentiostatic modes to form thin MIP film.	Direct electrode deposition; high sensitivity/selectivity; fast; ideal for sensors.	Only on conductive substrates; template removal tricky; thickness control challenging.	[217]
Microwave‐Assisted Polymerization	Standard polymerization mixture irradiated with microwaves for rapid, uniform thermal activation and polymer formation	Fast synthesis; homogeneous heat; energy‐efficient.	Requires microwave equipment; not easily scalable; risk of localized overheating.	[218]
Emulsion Polymerization	Monomer, template, cross‐linker, surfactant, initiator emulsified in water; polymerization in micelles yields nanoscale MIPs.	Nanoparticles with high surface area; controllable size; high binding capacity.	Surfactants may hinder recognition; complex purification; potential template leakage.	[219, 220]
Surface Imprinting	Template immobilized on substrate surface; monomers and cross‐linker polymerize around it; removal leaves accessible surface binding sites.	High binding‐site accessibility; fast kinetics; ideal for large biomolecules; selective.	Complex synthesis; lower overall binding capacity; requires precise surface functionalization.	[180]

For scalable nanoMIP production for clinical brain delivery, 1) solid‐phase (surface) imprinting, which is often coupled with controlled/RAFT kinetics, is recommended. This is because covalent template immobilization enables affinity‐based purification of high‐affinity, template‐free nanoMIPs with excellent batch‐to‐batch reproducibility and clean, ligand‐accessible surfaces that favor receptor‐mediated transcytosis and facilitate quality control, thereby supporting regulatory delineations.^[^ ^221^, ^222^ ^]^ 2) Miniemulsion/suspension polymerization (preferably surfactant‐free or employing reactive stabilizers), which offers tight control of clinically relevant particle sizes (≈50–200 nm), strong industrial scale‐up precedent, and proven continuous‐flow implementations that reduce variability, with emerging light‐driven surfactant‐free systems further lowering residual‐surfactant risk.^[^ ^223^ ^]^ 3) Precipitation polymerization (including RAFT/flow variants), which yields monodisperse, surfactant‐free particles suitable for straightforward postfunctionalization but typically requires larger solvent volumes and process intensification to achieve manufacturing‐scale yields.^[^ ^224^, ^225^ ^]^ Methods placed lower for injectable BBB applications include sol‐gel and electropolymerization.^[^ ^226^ ^]^ Thus, this ranking reflects (i) size control (miniemulsion/continuous‐flow as best, precipitation moderate, solid‐phase preserves core size), (ii) ease of functionalization (solid‐phase and precipitation provide cleaner surfaces), and (iii) regulatory feasibility (solid‐phase ensures consistent site presentation and enables robust purification; continuous heterophase routes have established scale‐up lineages; and surfactant‐free options reduce excipient risk), all of which are aligned with the need for reliable ligand display for receptor‐mediated brain delivery.

### 3.3 Bridging Synthesis to BBB Permeation Requirements

Precipitation polymerization reliably produces uniform, surfactant‐free nanoMIPs ≈50 to 100 nm in diameter with a low polydispersity index. This size range fits the typical BBB transport window and still leaves room for subsequent surface modification with targeting ligands. For example, a study reported the production of dipyridamole nanoMIPs with hydrodynamic sizes of ≈88 nm, which demonstrated high binding efficiency.^[^ ^163^ ^]^ Particles in this size range are well suited for RMT across the BBB, particularly when they display moderate affinity, often for monovalent ligands.^[^ ^113^, ^227^, ^228^, ^229^, ^230^, ^231^, ^232^ ^]^

Emulsion and miniemulsion polymerization offer precise size control in the ≈50–210 nm range with industrial scalability.^[^ ^166^, ^167^ ^]^ However, residual surfactants can alter the protein corona, obscure surface‐displaying targeting ligands, and thereby diminish RMT specificity while increasing nonspecific uptake.^[^ ^233^, ^234^ ^]^ To retain the size‐control advantages of emulsions while improving biocompatibility, two strategies are particularly effective: (i) surfactant‐free emulsion formulations and (ii) anchored or covalently bound surfactants that are integrated into the particle matrix to resist desorption.^[^ ^169^, ^170^, ^171^ ^]^ Therefore, the use of nanoMIPs to achieve efficient delivery at the BBB, quantitative assays for residual surfactant, proteomic characterization of hard or soft coronas, and ligand‐accessibility testing in serum are necessary to verify that targeting epitopes remain exposed under biologically relevant conditions.^[^ ^233^, ^234^ ^]^

Suspension polymerization can yield spherical nanoMIPs within the relevant size range of the BBB. For example, continuous‐flow microreactors have produced protein‐imprinted nanoMIPs of ≈50–100 nm within minutes with good monodispersity, and a microwave‐assisted suspension has similarly generated ≈120–140 nm particles on short timescales.^[^ ^134^, ^177^ ^]^ However, when stabilizers such as polyvinyl alcohols are employed, residuals must be rigorously minimized to preserve inherent characteristics and maintain ligand accessibility, thereby sustaining receptor‐mediated transport fidelity and limiting nonspecific uptake.^[^ ^233^, ^234^ ^]^ Therefore, quantitative assays for stabilizer carryover and protein‒corona profiling under serum conditions should be carried out to confirm the surface availability of the targeting motifs.

In surface (solid‐phase) imprinting, locating recognition sites in a thin outer shell increases their accessibility and accelerates target binding, which is advantageous for RMTs where the number of endothelial contacts is short.^[^ ^221^, ^235^, ^236^ ^]^ Solid‐phase methods also enable purification by exploiting the on‐bead affinity of suitably imprinted particles and improving batch‐to‐batch consistency. These properties align with TfR design principles that favor moderate‐affinity and monovalent formats to limit endothelial sequestration and lysosomal trafficking.^[^ ^113^, ^229^, ^230^ ^]^

Sol‒gel networks offer high porosity and robustness in nanoparticles, but injectable carriers require rigorous control and mechanical evaluation. As a result, they are usually a second‐choice option suitable for cases where specific inorganic functions are needed and the purification process is clinically validated.^[^ ^195^, ^196^, ^197^, ^198^, ^199^, ^200^ ^]^

Bulk imprinting and electrochemical deposition are useful for thin‐film sensors. However, their irregular morphologies lead to heterogeneous binding site distributions, and their processing constraints make them less suitable for circulating nanoMIPs, which require a narrow particle size distribution and ultraclean surfaces.^[^ ^156^ ^]^

From a synthesis‐to‐function perspective, precipitation and emulsion polymerizations offer complementary routes to low‐immunogenic nanoMIPs for BBB permeation. Precipitation polymerization is intrinsically surfactant‐free, enabling near‐neutral or slightly anionic, hydrophilic interfaces, readily achieved via PEGylation, that control opsonization and extend circulation; it can reproducibly yield monodisperse ≈50–100 nm particles with low PDI, dimensions consistent with BBB transport constraints.^[^ ^163^, ^231^, ^232^ ^]^ Emulsion or miniemulsion methods provide superior scalability and tight size control but can suffer from surfactant carryover that reshapes the protein corona, masks targeting ligands, and increases nonspecific uptake unless proactively managed; these risks are mitigated by surfactant‐free or reactive‐surfactant formulations together with rigorous quantification of residuals and desorption kinetics.^[^ ^166^, ^167^, ^169^, ^170^, ^171^, ^233^, ^234^ ^]^ From the TfR point of view, where moderate affinity and monovalent formats maximize brain exposure by avoiding endothelial sequestration, precipitation, or solid‐phase approaches often simplify precise ligand stoichiometry on clean surfaces, whereas emulsion‐derived systems should explicitly demonstrate that surfactant management preserves ligand accessibility in serum.^[^ ^229^, ^230^, ^233^, ^234^, ^237^ ^]^ To support cross‐study comparability, investigations should include dynamic light scattering in serum and surface charge targeted near‐neutral/slightly negative unless AMT is deliberately engaged. Quantitative assays for residual surfactant/initiator (e.g., HPLC/LC‒MS), protein‒corona proteomics coupled with ligand‒accessibility tests in 100% serum (SPR/ELISA or competitive binding), hemocompatibility/complement activation panels, and RMT‐relevant transcytosis assays (e.g., hCMEC/D3) with affinity tuning should also be evaluated.^[^ ^229^, ^230^, ^233^, ^234^, ^237^, ^238^ ^]^

### 3.4 Selection of Polymerization Routes for Designing BBB‐Permeable nanoMIPs

Method selection should prioritize the ability to reproducibly achieve (i) hydrodynamic diameters of ≈50–150 nm with low polydispersity, (ii) high accessibility of imprinted sites to support rapid association (fast on‐rates), (iii) minimal residual surfactant, initiator, and other reagents, and (iv) scalable, lot‐to‐lot reproducible manufacturing. These attributes can best predict in vivo BBB transport and consistent brain biodistribution.

#### 3.4.1 Solid‐Phase Surface Imprinting

For protein/peptide templates or epitope imprinting, where surface accessibility is prioritized, solid‐phase routes that immobilize the template and polymerize around it yield uniform, high‐affinity nanoparticles with built‐in affinity purification, improving batch uniformity and enabling automation; these protocols are now well established and reproducible. This is recommended when kinetic accessibility and template integrity in aqueous media are critical.^[^ ^221^, ^235^, ^236^ ^]^

#### 3.4.2 Precipitation Polymerization (Including Controlled/Living Polymerization)

For small‐molecule templates, precipitation polymerization reliably affords monodisperse, surfactant‐free particles and straightforward purification. RAFT‐enabled polymerization can further narrow the size distribution. The primary limitations are high solvent use and modest yields, which demand solvent recovery and/or flow‐assisted formats for scale‐up. This process is recommended when surfactant avoidance and narrow particle size distributions are prioritized.^[^ ^224^, ^225^, ^239^ ^]^

#### 3.4.3 Emulsion/Miniemulsion and Suspension (Including Inverse Formulations)

These heterophase routes are inherently scalable with excellent size control (often 50 ‐ 300 nm) and industrial precedent; however, surfactant selection and removal are critical to avoid bioincompatible residues. Reactive/anchored surfactants and rigorous purification mitigate this risk. This method is appropriate when large‐scale manufacturing and tight size control are needed, but additional quality assurance is needed to monitor and minimize residual surfactants.^[^ ^240^, ^241^ ^]^

#### 3.4.4 Microreactor/Microfluidic Processing (Cross‐Cutting)

Continuous, on‐chip synthesis enables minute‐scale production of bovine serum albumin–imprinted nanoparticles with tunable, narrowly distributed sizes (≈52–106 nm), improved reproducibility, and efficient reagent use. It addresses key scale‐up and batch‐to‐batch variability issues in conventional batch processing and is best applied to intensify precipitation or suspension workflows and stabilize lot‐to‐lot performance.^[^ ^134^ ^]^

#### 3.4.5 Sol‐gel and Hybrid Organic‒Inorganic Routes

Sol‐gel imprinting yields chemically and thermally robust matrices with controllable porosity and good aqueous compatibility, but it can pose challenges for template removal and may shrink or crack during drying. Hybrid organosilica shells can partially mitigate these issues. These materials are best suited for durable coatings or sensor‐grade interfaces. For in vivo colloidal carriers, brittleness and potential leachables/extractables must be validated on a case‐by‐case basis.^[^ ^200^, ^242^ ^]^

#### 3.4.6 Electrochemical Deposition

Electropolymerization is well suited for fabricating thin‐film MIPs directly on electrodes, enabling rapid, sensitive sensing. However, its utility for free, colloidal nanoparticles intended for systemic administration is intrinsically limited. Polymer growth is restricted to conductive substrates, and precise thickness control and complete template removal can be difficult to achieve. Accordingly, electropolymerization is best recommended for sensing interfaces rather than for injectable BBB carriers.^[^ ^243^ ^]^

In summary, for protein or epitope targets where rapid association kinetics are essential, prioritizing solid‐phase imprinting with immobilized templates, optionally coupled to RAFT control, and microreactor‐based intensification can be added to increase productivity. For small‐molecule targets and surfactant‐free particles, precipitation polymerization is preferred; RAFT‐mediated precipitation or continuous‐flow variants can further improve the particle size distribution and scalability, provided that solvent recovery plans are in place. When manufacturing scales and precise size control in the 50–200 nm range are the primary goals, miniemulsion or suspension routes using reactive surfactants, followed by stringent purification and quality control, are recommended; pairing these routes with microreactor platforms can reduce batch‐to‐batch variance. Finally, for coatings and sensor interfaces, sol‐gel or electropolymerized films are generally most suitable, whereas these approaches are not first‐line choices for developing injectable BBB‐crossing carriers.

### 3.5 Specificity and Selectivity through Tailored Recognition Sites

The hallmark of MIPs lies in their exceptional specificity and selectivity, which are precisely engineered through the creation of tailored recognition sites. This design principle fundamentally distinguishes MIPs as “synthetic antibodies” or “enzyme‐like receptors,” which are capable of binding target analytes with high affinity.^[^ ^244^, ^245^ ^]^ The core mechanism involves the formation of a highly specific three‐dimensional polymeric network around a template molecule, ensuring that the resulting cavities are perfectly complementary in size, shape, and chemical functionality to the target. The process begins with the careful selection of a template molecule, which can range from small molecules to complex biopolymers such as proteins and even entire cells.^[^ ^246^ ^]^ For example, studies have successfully created MIPs for proteins, which are crucial disease biomarkers and essential macromolecules involved in intra‐ and intercellular activities. The diverse functional groups and varying physicochemical characteristics (hydrophilicity, hydrophobicity, molecular grooves, and charges) present in proteins make them complex templates, yet MIP technology has advanced to effectively imprint them.

A critical step in achieving high specificity is the interaction between the template molecule and suitable functional monomers, leading to the formation of a “precomplex”.^[^ ^247^ ^]^ This precomplex is then polymerized in the presence of a cross‐linker, which locks the functional monomers into their positions relative to the template, forming a rigid polymer matrix. Computational methods, such as molecular docking and dynamics simulations, play crucial roles in predicting the preferred orientations and interactions between functional monomers (e.g., methacrylic acid (MA) and hydroxyethyl methacrylate (HEMA)) and target proteins (e.g., bovine serum albumin (BSA)).^[^ ^134^ ^]^ These simulations help in understanding the stability of the precomplex and the optimal stoichiometry of monomers for effective imprinting. For example, it has been shown that an MA‐HEMA dimer can exhibit double the interaction energy with BSA compared with individual MA‐BSA or HEMA‐BSA interactions, indicating its importance for continued polymerization.

Once the template is removed, the cavities formed within the polymer retain “molecular memory” of the template. These cavities possess specific binding sites that allow for highly selective rebinding of the target molecule. This selective binding is quantitatively assessed by parameters such as the selectivity coefficient (k) and the imprinting factor (k').^[^ ^248^ ^]^ A selectivity value greater than 1 indicates that the imprinted polymer is able to differentiate compounds; therefore, an imprinting effect is present. For example, BSA‐imprinted nanoparticles demonstrated a selectivity coefficient of 4.5 against human serum albumin (HSA), indicating their strong preference for BSA. The imprinting factor further elucidates the effect of molecular grooves on target binding, comparing the binding impact of MIPs versus NIPs. Studies have shown that imprinted nanoparticles can detect target proteins (e.g., BSA) with 2.3 times greater efficiency than NIP nanoparticles can, highlighting the critical role of specialized grooves.^[^ ^134^ ^]^ An IF value greater than 1 indicates good imprinting.^[^ ^249^ ^]^ The synthesis of nanoMIPs typically results in smaller particle sizes than those of NIPs, often because the template molecules can limit the growth of the polymer network during imprinting. This smaller size translates to a higher surface‐area‐to‐volume ratio, allowing for more interactions with target molecules and faster binding kinetics.^[^ ^131^ ^]^ The ability to precisely control the synthesis process, such as through microreactor systems, can further increase the uniformity and monodispersity of these nanoparticles, leading to more reproducible and efficient binding.^[^ ^134^ ^]^

In summary, the design principles of nanoMIPs, through appropriate template selection, judicious choice of functional monomers and cross‐linkers, and advanced polymerization techniques, culminate in materials with unparalleled specificity and selectivity. These tailored recognition sites are paramount for applications in highly demanding fields such as drug delivery, biosensing, and diagnostics.

### 3.6 Advantages of nanoMIPS Over Conventional Drug Carriers

nanoMIPs offer potential advantages that present them as superior alternatives to conventional drug carriers, especially in complex biomedical applications such as targeted drug delivery across the BBB. Their high affinity and selectivity, coupled with their robust physicochemical properties, address many limitations inherent in traditional delivery systems.

**Figure 6:**
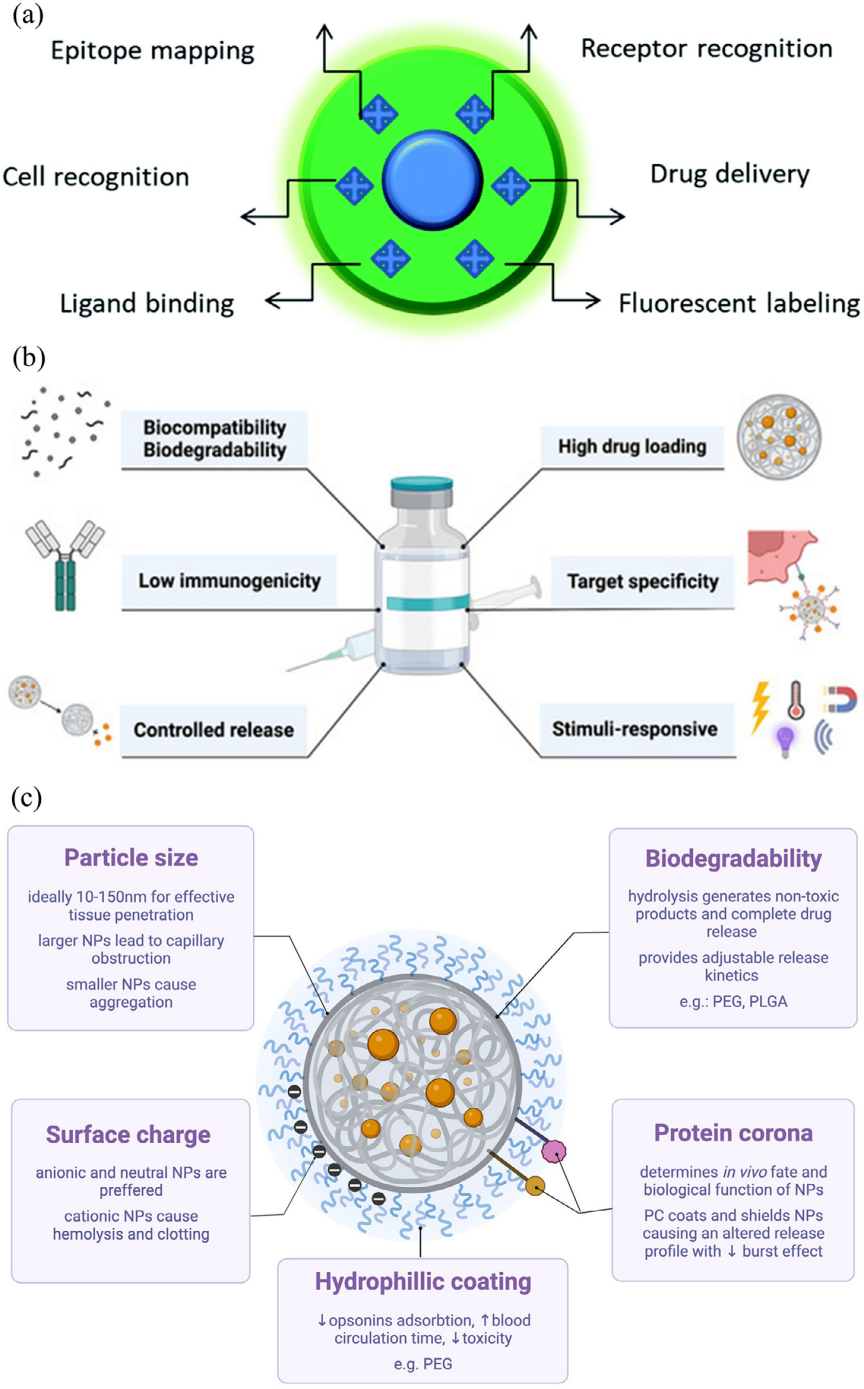
a) The advantages of nanoMIPs over traditional drug delivery carriers featuring biomedical applications depend on their composition and imprinting strategy, which prepares them to serve as versatile platforms for biosensing, targeted drug delivery, molecular therapy, cellular labeling, and epitope mapping. Reproduced with permission.^[^ ^250^ ^]^ Copyright 2022, Author(s). b) Key features of an ideal molecularly imprinted polymer (MIP) for drug delivery systems (DDSs). The illustration highlights critical attributes, including excellent biocompatibility, biodegradability, low immunogenicity, high drug‐loading capacity, controlled and stimuli‐responsive release, and precise target specificity. Reproduced with permission.^[^ ^251^ ^]^ Copyright 2022, Author(s). c) Factors influencing the biocompatibility of nanoparticles include size, surface charge, hydrophilic surface modifications, biodegradability, and the formation of a protein corona. Abbreviations: PEG, poly(ethylene glycol); PLGA, poly(lactic‐co‐glycolic acid); PC, protein corona; NP, nanoparticle. Reproduced with permission.^[^ ^251^ ^]^ Copyright 2022, Author(s).

By mimicking natural ligands, nanoMIPs can enable the precise recognition of cell surface markers, allowing the selective targeting of specific cell populations. Their imprinted structures facilitate efficient drug loading and controlled release, making them ideal for targeted therapy. In addition to drug delivery, nanoMIPs serve as powerful platforms for biosensing, cellular labeling, and epitope mapping. For example, nanoMIPs imprinted against specific proteins can protect bound epitopes from enzymatic digestion, enabling rapid identification of these regions. Moreover, nanoMIPs designed against cell surface receptors can modulate cellular physiology by blocking ligand‒receptor interactions (**Figure** **6**a,b).^[^ ^250^, ^251^ ^]^

An emerging aspect in the evaluation of MIP biocompatibility is the role of the protein corona, which forms when nanoparticles interact with biological fluids.^[^ ^252^, ^253^ ^]^ Increasing evidence indicates that the in vivo fate, biodistribution, and therapeutic efficacy of nanoMIPs are strongly influenced by corona formation. Consequently, any biomedical application of MIPs should carefully consider the dynamics and composition of the protein corona. Recent studies suggest that the nature of the protein corona is a critical determinant of nanoparticle clearance from the body, indicating the need for further investigation. The adsorption of proteins onto the MIP surface can coat and shield the nanocarrier, potentially altering its drug release kinetics. For example, protein corona formation may substantially reduce the initial burst release by limiting the desorption of surface‐bound drugs. Nevertheless, some reports on polymeric nanoparticles indicate that the influence of the protein corona on release profiles can be minimal, suggesting that the impact is material‐ and context‐dependent. A schematic overview of the principal factors influencing MIP biocompatibility, including PC formation, is summarized in Figure 6c.^[^ ^251^ ^]^

One of the most significant advantages of nanoMIPs is their high affinity and selectivity for target molecules, which mimics the “lock and key” principle of natural antibodies.^[^ ^250^ ^]^ Unlike conventional carriers, which often rely on nonspecific interactions or general targeting mechanisms, nanoMIPs are engineered with tailor‐made recognition sites that bind precisely to their specific target, whether it is a drug, a protein, or a disease biomarker. This specificity is crucial for targeted drug delivery, ensuring that the therapeutic payload reaches the intended site with minimal off‐target effects and increased therapeutic efficacy. Here, the precise molecular grooves created in MIPs led to significantly greater binding efficiency for target proteins than nonimprinted polymers.

Another critical advantage is the exceptional stability and robustness of MIPs.^[^ ^254^ ^]^ Unlike biological receptors, which are susceptible to denaturation under various pH values, temperatures, and enzymatic degradation conditions, MIPs are synthesized as highly cross‐linked polymeric structures that exhibit remarkable resistance to harsh conditions. This intrinsic stability allows for a longer shelf‐life, easier storage (often at room temperature), and greater reusability, reducing the logistical complexities and costs associated with temperature‐sensitive biological agents.

NanoMIPs also boast superior preparation and cost‐effectiveness compared with traditional drug carriers, particularly biological antibodies.^[^ ^255^ ^]^ The synthesis of MIPs can be less time‐consuming and labor‐intensive, and their production cost is significantly lower than that of antibodies. Recent advancements in microreactor technology enable the continuous synthesis of MIP nanoparticles in a fraction of the time (5–30 minutes compared with 24 hours for conventional bulk methods), with higher yields and improved reproducibility. This innovative approach can also minimize reagent volumes and reduce overall production costs significantly, potentially down to a cost‐effective price point per chip.^[^ ^134^ ^]^ Recently, Saczek et al. reported the use of nanoMIPs for sensing the troponin I biomarker in clinical samples, indicating promising advancements in cardiovascular disease diagnostics and treatment.^[^ ^256^ ^]^

Furthermore, the tunable size and high surface‐area‐to‐volume ratio of nanoMIPs increase their utility in biomedical applications. With diameters typically less than 200 nm, nanoMIPs offer rapid binding kinetics, good dispersion properties, and ease of handling.^[^ ^257^ ^]^ This high surface area allows them to interact with a greater number of target molecules, which is crucial for efficient drug loading and delivery. Moreover, their small size is advantageous for navigating complex biological environments and potentially crossing biological barriers such as the BBB.^[^ ^258^ ^]^

NanoMIPs also offer ease of functionalization and surface modification, which are essential for targeted drug delivery and overcoming biological barriers. Their polymeric nature allows various chemical modifications to incorporate targeting ligands, surface modifications, or stimuli‐responsive elements, further enhancing their capabilities. This versatility allows for the design of “smart” drug delivery systems that can respond to specific environmental stimuli, such as pH changes or the presence of certain molecules, leading to controlled and prolonged drug release.^[^ ^259^ ^]^

Through this surface modification, nanoMIPs can easily cross the BBB. In other words, nanoMIPs can utilize endogenous transport mechanisms, cellular hitchhiking, and stimuli‑responsive release systems through chemical modification. The advantages of the ability of nanoMIPs to pass through the BBB will be explained in detail in later sections.

Finally, the ease of template removal and the resulting homogeneous binding sites contribute to their effectiveness. This ensures that the generated cavities are readily available for rebinding the target molecule and minimizes nonspecific binding, which can be a common issue with some conventional carriers.^[^ ^131^ ^]^ In summary, the combination of high specificity, exceptional stability, cost‐effective and scalable production, tunable physical properties, and versatile functionalization makes nanoMIPs promising and transformative platforms for advanced drug delivery, offering significant improvements over conventional carrier systems for addressing challenging therapeutic targets such as the brain.

### 3.7 Stability, Biocompatibility, and Binding Affinity of nanoMIPs

The therapeutic applicability of nanoMIPs, especially for permeating the BBB, is critically dependent on their physicochemical stability, biocompatibility, and finely tuned binding affinity. These fundamental characteristics dictate their effectiveness, safety, and ultimate clinical translatability. Stability is an inherent and highly advantageous feature of MIPs, setting them apart from many biological recognition elements such as antibodies or enzymes. Unlike biomolecules, which are often sensitive to denaturation by extreme temperatures, pH variations, or enzymatic degradation, MIPs are synthesized as highly cross‐linked polymeric structures that thereby have excellent stability.^[^ ^260^ ^]^ This structural property allows MIPs to maintain their specific recognition properties under a wide range of harsh conditions, making them ideal for long‐term storage and reuse without significant loss of function.^[^ ^254^, ^261^ ^]^ For example, Smolinska‐Kempisty et al. reported that nanoMIPs imprinted against biotin demonstrated remarkable stability, maintaining their original detection limit even after a month.^[^ ^262^ ^]^ This sustained performance over an extended period indicates that the biotin‐imprinted nanoMIPs are suitable for scenarios requiring prolonged storage or repeated use without damaging their sensitivity. The ability of these methods to retain consistent detection capabilities makes them highly promising for practical applications where stability is a critical factor. In a study by Melemdez‐Marmolejo et al., MIPs displayed high thermal stability at temperatures up to 300 °C.^[^ ^263^ ^]^​ Here, the presence of 2‐vinylpyridine as a functional monomer was observed to enhance the thermal stability of the material even further. This thermal and chemical stability also simplifies their sterilization and handling, which is crucial for pharmaceutical applications. The choice of monomers and cross‐linkers, such as EGDMA, can contribute to the rigidity and stability of the polymeric network.^[^ ^264^ ^]^

Biocompatibility is an important factor for any material intended for in vivo administration. This refers to the ability of a material to perform its intended function without causing undesirable local or systemic adverse effects in the biological environment.^[^ ^137^, ^265^, ^266^ ^]^ While MIPs are synthetic, careful selection of monomers and optimization of synthesis conditions are essential to minimize toxicity and immunogenicity. Studies on the biocompatibility of polymeric nanoparticles, including those used in MIP synthesis, have explored their internalization by cells and potential cytotoxicity. For example, PLGA‐PEG nanoparticles at concentrations up to 50 µg mL^−1^ maintained 80% viability in human brain microvascular endothelial cells (hCMEC/D3), indicating good in vitro biocompatibility.^[^ ^267^ ^]^ Furthermore, some polymers, such as polypyrrole, exhibit notable compatibility with biological entities and do not irritate the mammalian immune system, suggesting their potential for use as implantable biosensors.^[^ ^268^ ^]^ In vivo assessments are crucial for understanding the biodistribution, clearance, and potential systemic toxicity of nanoMIPs. Initial in vivo studies, such as those in rat models, have investigated these aspects, indicating progress toward understanding their safety profile. However, further extensive in vivo studies are still needed to fully validate their long‐term safety and biocompatibility for human applications.^[^ ^269^ ^]^

Binding affinity is another important factor that can enhance the efficacy of MIPs as drug carriers, as it determines how strongly and specifically the MIP can bind to its target molecule. High affinity ensures efficient drug loading and targeted delivery, whereas high selectivity minimizes off‐target binding, which can lead to reduced therapeutic effects and increased side effects.^[^ ^250^ ^]^ The molecular imprinting process is designed to create optimal binding sites that are complementary in shape and chemical functionality to the template molecule. The choice of functional monomers, their stoichiometric ratio to the template, and the polymerization conditions significantly influence the number and quality of these binding sites.^[^ ^264^, ^270^ ^]^ Evaluation of binding affinity typically involves equilibrium adsorption experiments, which can quantify the density of binding sites and their dissociation constants. The “imprinting factor” (k′), which compares the binding capacity of MIPs to that of NIPs, directly reflects the contribution of the specific recognition sites. Studies have demonstrated that MIPs consistently exhibit higher affinity and recognition for their target molecules than NIPs do, often detecting them with several times greater efficiency. For example, BSA‐imprinted nanoparticles were found to detect BSA proteins with several times greater efficiency than NIPs did, highlighting the success in creating specific binding sites.^[^ ^134^, ^271^ ^]^ Precise control over nanoparticle synthesis, such as through microreactor systems, can lead to more uniform structures and smaller particle sizes, which in turn increase the surface area for binding and improve the overall binding capacity and reproducibility. Therefore, the ability of MIPs to bind selectively to target molecules and release them in a controlled manner is a key factor.^[^ ^272^ ^]^

In summary, the inherent stability of MIPs provides a robust foundation for their use as drug carriers. While biocompatibility requires ongoing rigorous in vitro and in vivo evaluation, the promising initial results reported thus far suggest the significant potential of these materials. The accurately engineered binding affinity, which is critical for targeted delivery and therapeutic efficacy, is a core strength of molecular imprinting technology, collectively positioning nanoMIPs as a viable and intelligent platform for next‐generation brain therapies.

## 4 nanoMIPs for BBB Permeation

The design and fabrication of nanoMIPs for delivery across the BBB necessitate a multifaceted approach, encompassing template selection, choice of functional monomers, polymerization strategies, and optimization of the physicochemical properties of the nanocarrier. The principal target is to create “synthetic antibodies”, also known as “plastic antibodies” with high affinity and selectivity, capable of navigating the complex microenvironment of the brain.^[^ ^134^ ^]^

### 4.1 Selection of Templates Relevant to Brain‐targeting Therapeutics

The initial and perhaps most critical step in designing MIP nanocarriers for brain‐targeting therapeutics is the selection of appropriate template molecules.^[^ ^272^ ^]^ The template essentially dictates the “molecular memory” of the polymer, shaping the specific recognition sites that ultimately bind the therapeutic agent or a biomarker relevant to brain diseases. Templates can range from small drug molecules to complex biomacromolecules such as proteins or peptides, and even entire cells.^[^ ^273^ ^]^ For brain disease therapy, relevant templates might include neurotransmitters and specific neurotoxic proteins implicated in neurodegenerative diseases, e.g., *β*‐amyloid for Alzheimer's disease, *α*‐synuclein for Parkinson's disease, components of brain tumors, or even viral proteins for CNS infections.^[^ ^274^, ^275^ ^]^

When large biomolecules such as proteins, which are critical disease biomarkers involved in various cellular activities, are imprinted, the challenge lies in their complex structures with various functional groups, hydrophilicity, hydrophobicity, and molecular grooves.^[^ ^272^, ^276^ ^]^ Advanced techniques such as boronate affinity‐based molecular imprinting, solid‐phase synthesis, and postimprinting modification have been developed to address these complexities, enabling the precise and selective isolation of proteins from complex biological samples for diagnostic and therapeutic applications.^[^ ^209^, ^277^, ^278^, ^279^ ^]^ The solid‐phase synthesis strategy allows for MIP preparation specifically for proteins that can be immobilized in an oriented way on a solid support, enhancing reproducibility and automation.^[^ ^187^, ^280^ ^]^ The selection of a template directly influences the binding affinity and specificity of the resulting MIPs, making it a foundational decision in the design process.^[^ ^281^ ^]^

### 4.2 Functional Monomers and Cross‐Linkers for Imprinting

The selection of functional monomers and cross‐linkers is fundamental to the architecture of MIPs, as these components govern the formation of specific recognition sites and dictate interactions with the template molecule. Functional monomers are chosen on the basis of their ability to form stable prepolymerization complexes with the template through diverse noncovalent interactions, including hydrogen bonding, ionic forces, π‒π stacking, ion‒dipole interactions, hydrophobic effects, and van der Waals forces.^[^ ^282^, ^283^ ^]^ For example, methacrylic acid (MAA) and 2‐vinylpyridine (2‐VP) have been widely investigated, and their interactions with target proteins such as bovine serum albumin (BSA) have been extensively studied via molecular docking and molecular dynamics simulations.^[^ ^134^, ^150^, ^284^ ^]^ Such computational approaches provide critical insights into the optimal orientations and interaction energies between monomers and templates, thereby guiding the rational design of highly stable precomplexes and enabling the selection of suitable monomers for specific targets.

**Scheme 1:**
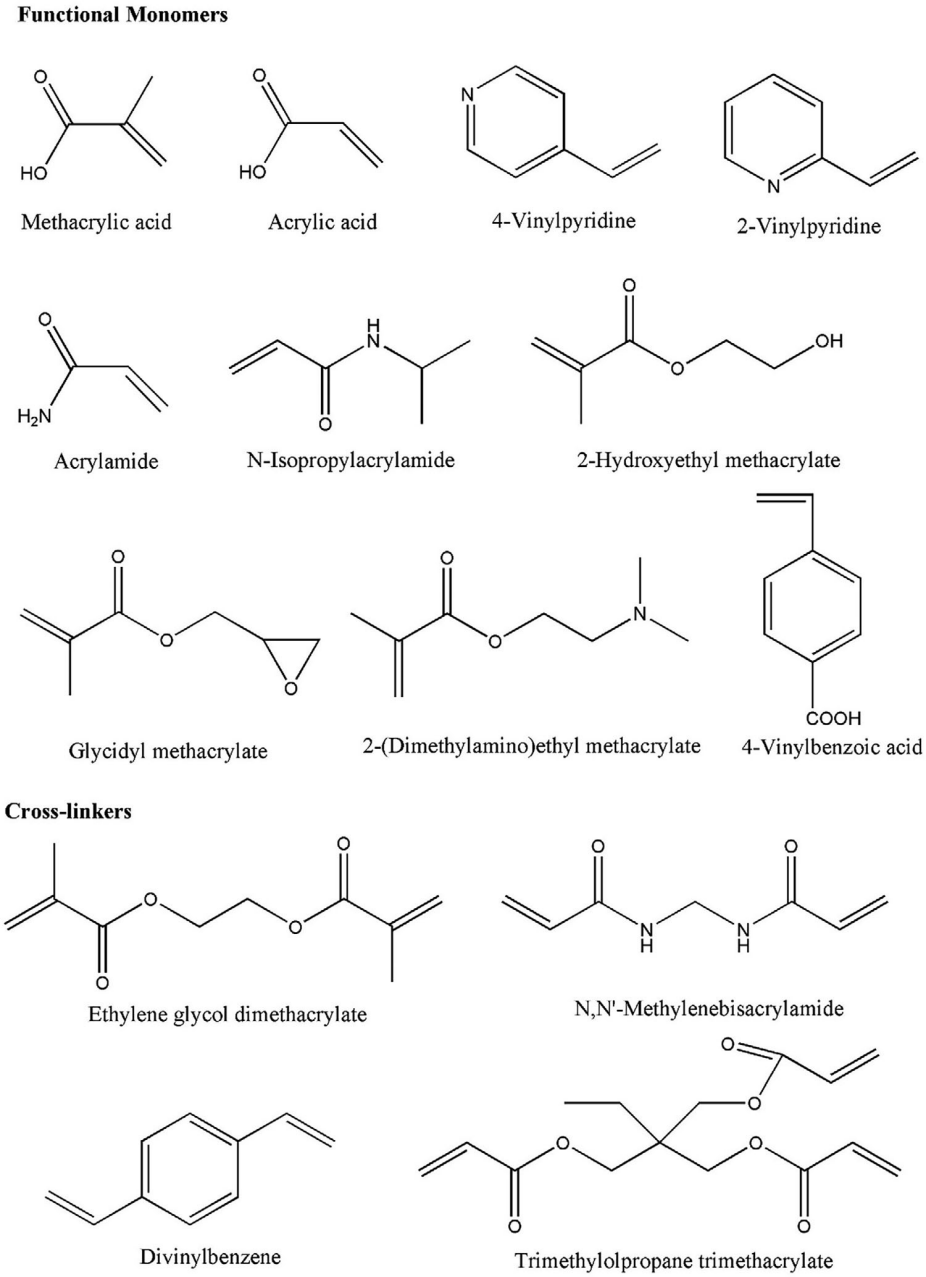
Representative examples of functional monomers and cross‐linkers commonly employed in the synthesis of molecularly imprinted polymers (MIPs).

On the other hand, cross‐linkers provide mechanical stability to the polymer matrix and preserve the geometry of the imprinted cavities after template removal.^[^ ^282^, ^285^ ^]^ EGDMA is considered the gold standard cross‐linker owing to its ability to form rigid, highly cross‐linked polymer networks.^[^ ^286^ ^]^ The ratio of functional monomers to cross‐linkers is a critical determinant of the binding site density, accessibility, and overall structural integrity of a polymer. For example, a commonly used template:functional monomer:cross‐linker ratio of 1:4:20 has been shown to yield optimized binding performance.^[^ ^287^ ^]^ Additionally, the choice of polymerization solvent or “porogen” strongly influences the morphology and porosity of the final polymer, which in turn affects both the binding capacity and release kinetics.^[^ ^135^, ^288^ ^]^ Recently, deep eutectic solvents (DESs) have gained attention both as porogens and as functional monomers because of their ability to increase binding selectivity and affinity while promoting greener and more sustainable imprinting strategies. For example, Han et al. developed MOF@DES‐MIPs capable of selectively recognizing bovine hemoglobin, where the DES facilitated multiple interaction forces during imprinting, thereby strengthening H‐bonding and electrostatic interactions.^[^ ^289^ ^]^ Similarly, He et al. reported DES‐enabled protein imprinting using *β*‐hydroxybutyrate, further supporting the potential of DESs to enhance noncovalent interactions.^[^ ^290^ ^]^ Consistent with these findings, several other studies have demonstrated improved imprinting performance and binding capacity for diverse analytes, such as parabens and bisphenol A.^[^ ^291^, ^292^ ^]^ Representative examples of commonly used functional monomers and cross‐linkers that form the structural basis of MIPs are presented in **Scheme** **1**.

Owing to these advances in monomer and cross‐linker design, nanoMIPs have recently emerged as promising platforms for drug delivery across or toward the BBB. For example, rivastigmine tartrate‐imprinted nanoparticles were synthesized via precipitation polymerization using poly(methacrylic acid) (poly‐MAA) as the functional monomer and EGDMA as the cross‐linker. These nanoparticles exhibited sustained and controlled in vitro release, suggesting their suitability for central nervous system (CNS) drug delivery, although their BBB permeability was not directly assessed.^[^ ^293^ ^]^ In a more advanced approach, apolipoprotein (ApoE)‐conjugated nano‐MIPs have been designed for donepezil delivery to actively enhance BBB penetration. Following nano‐MIP fabrication, ApoE was conjugated to the surface to exploit receptor‐mediated transcytosis across BBB endothelial cells. In vitro studies using the hCMEC/D3 human BBB model demonstrated an ≈1.9‐fold increase in permeability compared with that of non‐ApoE‐modified MIPs, whereas in vivo investigations confirmed sustained delivery of donepezil to the brain.^[^ ^294^ ^]^ Therefore, these studies highlight how judicious selection of monomers, cross‐linkers, porogens, and targeting ligands can be strategically combined to produce highly selective MIPs with tailored physicochemical properties and enhanced therapeutic efficacy for CNS‐related drug delivery. The key functional monomers and cross‐linkers used to tailor MIP matrices for specific recognition properties, highlighting their structural features, interaction mechanisms with template molecules, and role in achieving high selectivity, strong binding affinity, and controlled release performance in MIP‐based applications, are summarized in **Table** **3**.

**Table 3:** Representative functional monomers and cross‐linkers commonly employed in the design and fabrication of MIPs.

Molecules	Role in MIP	Applications
Monomers		
Methacrylic acid	Acidic H‐bond donor/acceptor	Widely used for basic drugs; provides strong hydrogen bonding sites.
Acrylic acid	Acidic H‐bond donor/acceptor	Preferred in aqueous MIP formulations due to better solubility.
4‐Vinylpyridine	Basic H‐bond acceptor	Suitable for acidic templates; enhances selective binding.
2‐Vinylpyridine	Basic H‐bond acceptor	Similar to 4‐VP but offers different binding orientation.
Acrylamide	Neutral H‐bond donor/acceptor	Excellent for aqueous systems; often used with N,N′‐Methylenebisacrylamide cross‐linker.
N‐Isopropylacrylamide	Thermoresponsive monomer	Adds temperature‐sensitive drug release properties.
2‐Hydroxyethyl methacrylate	Hydrophilic, H‐bond donor	Common in hydrogel‐based MIPs for biomedical applications.
Glycidyl methacrylate	Epoxy‐functional monomer	Enables postimprinting modifications and conjugations
2‐(Dimethylamino)ethyl methacrylate	Cationic, pH‐responsive	Used for pH‐sensitive MIPs and enhanced drug release control.
4‐Vinylbenzoic acid	Aromatic acidic monomer	Combines π‐π interactions with H‐bonding for aromatic drugs.
Cross‐linkers		
Ethylene glycol dimethacrylate		Widely used cross‐linker; ensures rigid cavity formation and good porosity.
N,N'‐Methylenebisacrylamide		Hydrophilic cross‐linker; commonly used with acrylamide‐based systems.
Divinylbenzene		Produces highly rigid aromatic MIPs; useful for nonpolar templates.
Trimethylolpropane trimethacrylate		Provides very high cross‐link density, enhancing mechanical strength.

### 4.3 Size, Morphology, and Surface Chemistry Optimization for BBB Permeation

The success of nanoMIPs in overcoming the BBB is heavily dependent on the optimization of their size, morphology, and surface chemistry. Size is a critical determinant of BBB penetration. Generally, smaller nanoparticles exhibit better brain penetration.^[^ ^295^, ^296^, ^297^ ^]^ Studies have shown that polymeric nanoparticles with mean sizes around or below 160 nm can be internalized by brain endothelial cells in vitro.^[^ ^2^, ^258^, ^298^ ^]^ Microreactor synthesis has enabled the production of nanoMIPs with diameters as small as 52–106 nm, which is highly advantageous for BBB permeation because of the increased surface area for interaction.^[^ ^134^ ^]^ Shilo et al. reported the influence of nanoparticle size on BBB transport via barbiturate‐coated gold nanoparticles (GNPs) and an in vitro bEnd.3 endothelial cell model.^[^ ^299^ ^]^ The results indicated that 70 nm GNPs achieved the highest intracellular gold content, whereas 20 nm GNPs provided the greatest free surface area. These findings highlight particle size as a critical factor in optimizing nanoMIP design for brain‐targeted imaging and therapeutic applications. Therefore, the ability to precisely control the particle size and achieve monodispersity is vital for consistent in vivo performance and reproducible brain delivery.

Morphology also plays a role, with spherical nanoparticles often preferred for their ease of synthesis and favorable biodistribution.^[^ ^300^, ^301^ ^]^ Hollow porous or concave molecularly imprinted structures have been shown to significantly enhance the adsorption capacity and binding kinetics. For example, hollow porous dummy MIPs have very high surface areas (≈544 m^2^ g^−1^) and fast binding (equilibrium in ≈25 min), which is attributed directly to their porous morphology.^[^ ^302^ ^]^ Similarly, hollow porous MIP nanospheres prepared via mesoporous silica templates presented faster adsorption rates and higher affinities than conventional polymers did, confirming the advantages of engineered porosity and a high surface area.^[^ ^303^ ^]^ Although spherical nanocarriers have received considerable attention because of their favorable circulation dynamics, alternative morphologies are increasingly needed. Nonspherical architectures show promise for achieving slower clearance and prolonged retention at the BBB, potentially increasing local drug concentrations and therapeutic efficacy​.^[^ ^304^ ^]^ For example, worm‐like pH‐responsive mPEG‐b‐PDPA micelles with a diameter of 20 nm and length of 60–600 nm were engineered by Zeng et al. to deliver RGD peptide‐emtansine conjugates (RGD‐DM1) for brain tumor targeting.^[^ ^305^ ^]^ Upon intracellular acidification, the nanoworms dissociate to release RGD‐DM1, which is subsequently cleaved to DM1, enabling deep tumor penetration and efficient glioma cell uptake. In an orthotopic brain tumor model. This strategy achieved nearly 90% inhibition of tumor progression, highlighting its potential as an effective brain‐targeted therapy.^[^ ^304^ ^]^ Currently, research in this domain remains limited, necessitating systematic studies to fully elucidate the influence of nanoMIP morphology on BBB transport and brain‐targeted drug delivery.

Surface chemistry is arguably the most important factor for enhancing BBB transport. Plain polymeric nanoMIPs often struggle to cross the BBB efficiently. Surface modifications are therefore essential to facilitate their passage.^[^ ^227^, ^306^ ^]^ This includes the incorporation of targeting ligands. Conjugating specific ligands or antibodies to the nanoparticle surface can exploit endogenous BBB transport mechanisms, such as RMT. Ligands targeting receptors such as TfRs, insulin receptors, or LDL receptors can enhance transport into the brain. For example, ligand‐coated fluorescent TEB‐based nanoparticles demonstrated high BBB transport efficiencies (up to 29.02%) in an in vitro BBB model.^[^ ^227^ ^]^ Again, recent advances have focused on ligand‐modified nanomedicines, such as PLGA nanoparticles conjugated with the BBB‐penetrating peptide angiopep‐2 (Ang‐2). A study by Hoyos‐Ceballos et al. demonstrated that these sub200 nm nanoparticles successfully crossed the BBB and accumulated in neuronal cells, with in vivo analyses confirming their localization in brain regions, including the cortex and hippocampus.^[^ ^228^ ^]^ These findings highlight that Ang‐2‐modified PLGA nanoMIPS can be designed as a promising platform for brain‐targeted drug delivery. However, challenges remain in optimizing transcytosis versus lysosomal degradation.

#### 4.3.1 Balancing ligand Affinity to Avoid Lysosomal Trapping

For RMT targets such as TfRs, there is a narrow design window in which ligand density, valence, and apparent affinity promote transcytosis rather than endothelial retention and lysosomal routing. High‐affinity or bivalent binders can crosslink TfR and bias sorting to lysosomes with concentration‐dependent receptor downregulation, yielding the characteristic bell‐shaped exposure and affinity relationship; conversely, monovalent and lower‐affinity formats improve parenchymal exposure by minimizing endothelial sequestration.^[^ ^113^, ^229^, ^230^, ^237^ ^]^ This tradeoff, established for TfR‐shuttled proteins and nanoparticles, should be carefully considered when presenting nanoMIP ligands on the surface, such as areal density, accessibility above the corona, and monovalent display.

#### 4.3.2 Protein Corona Interference with Ligand Function

In plasma, the protein corona can form within seconds, which can shield targeting ligands and prevent receptor recognition; however, cellular uptake may still occur via nonspecific pathways. This phenomenon has been directly demonstrated for transferrin‐decorated nanoparticles and is broadly observed across various nanomaterials.^[^ ^233^, ^234^, ^307^, ^308^ ^]^ Therefore, the composition of the corona, for example, enrichment with dysopsonins such as clusterin, can reduce cellular uptake. Nevertheless, when suitably engineered, these interactions can be exploited to promote specific biological responses. This finding highlights the importance of reporting both ligand density and accessibility after corona formation via assays such as protease protection or competitive binding in serum so that the targeting performance can be accurately interpreted.^[^ ^234^, ^308^ ^]^

Polymers such as PEG are often used to coat nanoparticles (PEGylation), which helps reduce opsonization and clearance by the reticuloendothelial system (RES), thereby prolonging the circulation time in the bloodstream.^[^ ^231^, ^232^ ^]^ This property enhances the chances of nanoparticles reaching the BBB. The surface charge of nanoparticles influences their interaction with the negatively charged glycocalyx of brain endothelial cells, as reported by Olivieri Jr et al.^[^ ^238^ ^]^ Cationic molecules or nanoparticles can trigger AMT through electrostatic interactions, leading to nonspecific endocytosis. However, nonspecificity can lead to widespread tissue distribution and potential toxicity, highlighting the need for careful charge optimization. Therefore, careful tuning of the surface charge is essential to balance the uptake efficiency with specificity and safety.

Biomimetic strategies that incorporate elements that mimic natural transport pathways or cellular behaviors can be highly effective. For example, molecularly imprinted nanogels can acquire biocompatibility in situ by adjusting themselves with native dysopsonic proteins such as albumin, prolonging their circulation and enhancing their accumulation in the target. Another emerging approach involves cellular hitchhiking, where nanoparticles are loaded onto or associated with immune cells such as neutrophils, leveraging their natural ability to cross the BBB.^[^ ^309^ ^]^

**Figure 7:**
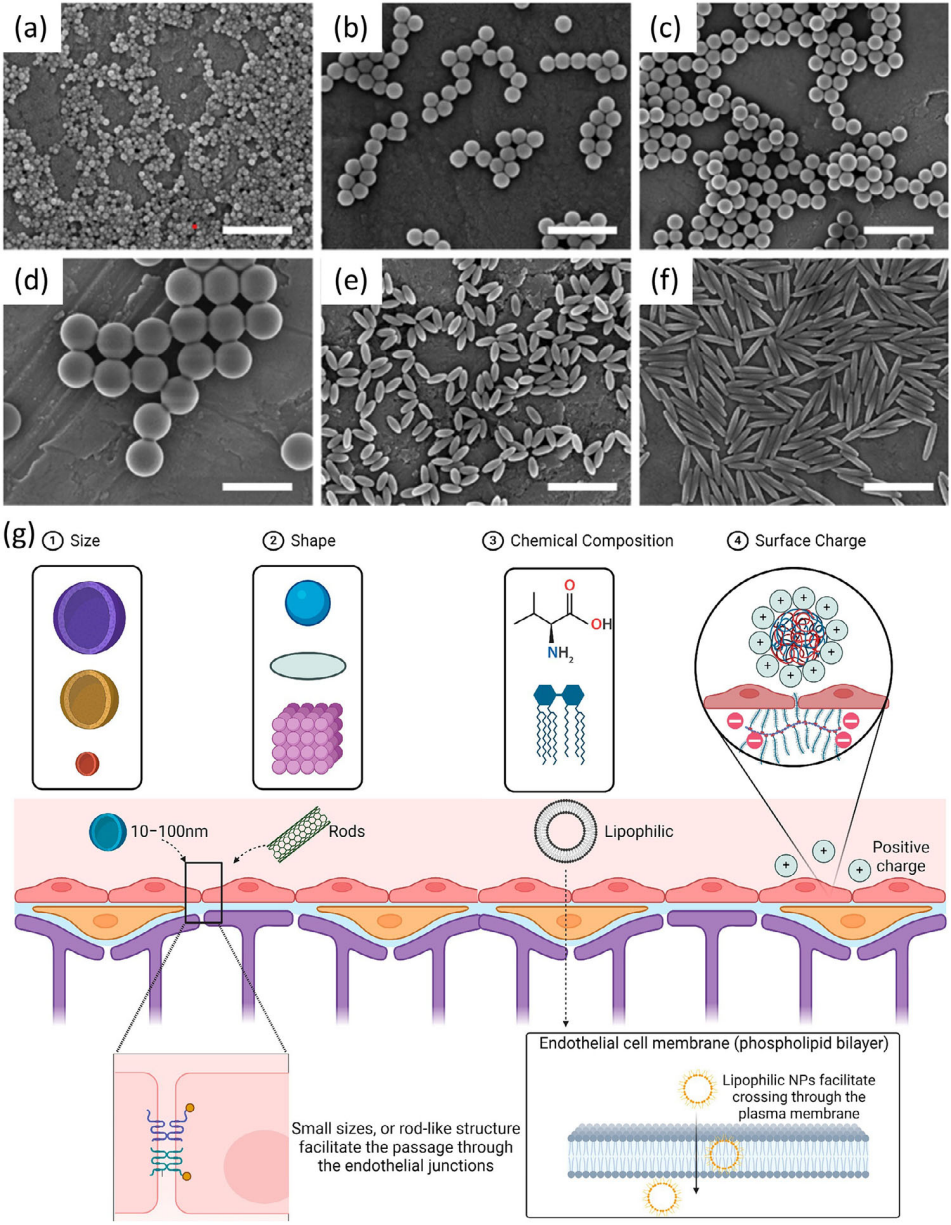
The effects of nanoparticle composition, size, shape, and stiffness on penetration across the BBB revealed that protein particles with a size of 500 nm whose surface was modified by transferrin showed better permeation. Scanning electron micrographs of nanoparticles: a) 50 nm polystyrene (PS) sphere (50‐PS), b) 100 nm PS, sphere (100‐PS), c) 200 nm PS sphere (200‐PS), d) 500 nm PS sphere (500‐PS), e) 2AR PS rod (2AR‐PS‐R), f) 5AR PS rod (5AR‐PS‐R). a–f) Reproduced with permission.^[^ ^310^ ^]^ Copyright 2020, ACS. g) Influence of physicochemical properties on nanoparticle‐mediated BBB penetration. Size, shape, chemical composition, and surface charge are key determinants of transport efficiency, with small, rod‐shaped, lipophilic, and positively charged nanoparticles exhibiting a superior ability to cross BBB endothelial junctions. Reproduced with permission.^[^ ^311^ ^]^ Copyright 2024, Author(s).

In a recent study, Brown et al. presented a library of nanoparticles of various sizes, shapes, stiffnesses, surface chemistries, and compositions along with their systematic evaluation via an in vitro human BBB model based on hCMEC/D3 cells.^[^ ^310^ ^]^ Analyses of particle uptake, apparent permeability, and endocytic pathways revealed that particle composition had the most significant influence on BBB penetration. Among the tested formulations, protein‐based particles with a size of 500 nm whose surface was functionalized by transferrin demonstrated the highest uptake and transport, likely mediated by a specific internalization mechanism (**Figure** **7**a–f). These findings provide critical insights into the design of nanoparticle‐based drug delivery systems and highlight promising opportunities for developing brain‐targeted therapeutics through rational engineering of particle properties. In a study by Asimakidou et al., small nanoparticles with a rod‐like structure, which are lipophilic in nature and bearing positive charges, had a greater ability to cross BBB endothelial junctions (Figure 7g).^[^ ^311^ ^]^ In one of our earlier studies, we similarly observed that nanoparticles with a higher aspect ratio exhibited superior potential for BBB permeation, likely due to their enhanced retention and interaction dynamics under flow conditions compared with their spherical counterparts.^[^ ^2^ ^]^ Thus, the meticulous design and fabrication of MIP‐based nanocarriers, from template selection to surface modification and precise control over particle characteristics, are pivotal for overcoming the BBB and realizing their full therapeutic potential in treating brain diseases. The synergy between molecular imprinting technology and nanotechnology continues to open new avenues for intelligent and targeted drug delivery to the CNS.

## 5 Strategies to Increase BBB Transport via nanoMIPs

### 5.1 Endogenous BBB Transport Mechanisms

**Figure 8:**
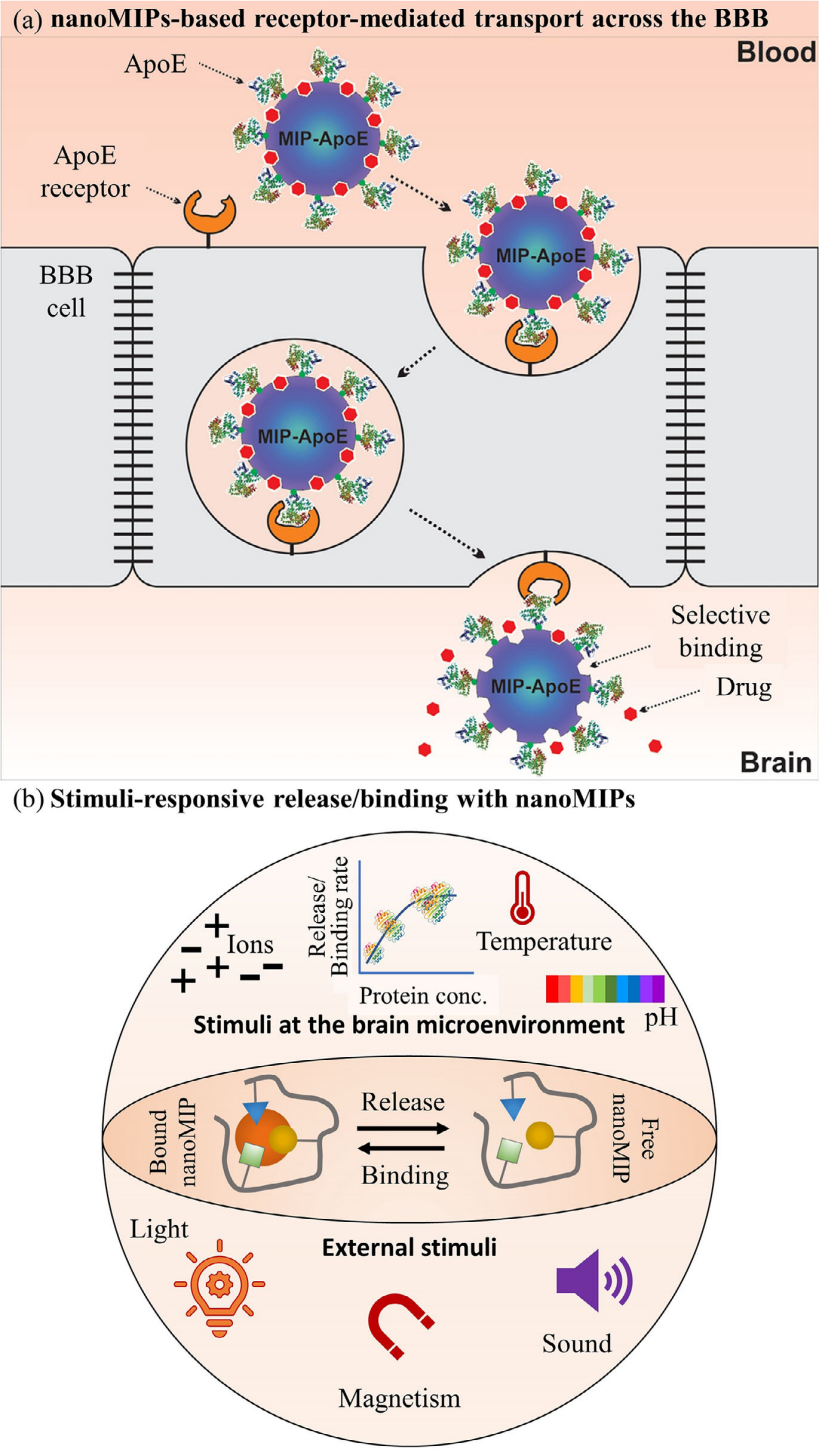
a) Schematic illustration of ApoE‐functionalized nanoMIPs enabling receptor‐mediated transport (RMT) across the BBB. Surface functionalization with ApoE facilitates receptor‐specific targeting and codelivery of therapeutic payloads, thereby increasing BBB permeability and transcytosis efficiency. Reproduced with permission.^[^ ^294^ ^]^ Copyright 2023, Springer Nature. b) A stimuli‐responsive nanoMIP designed for controlled drug/template release and binding. Internal triggers in the brain microenvironment (e.g., pH, temperature, ion concentration, and protein levels) and external, noninvasive stimuli (e.g., light, magnetic fields, and sound) induce conformational and physicochemical changes within the MIP matrix, enabling precise, on‐demand therapeutic payload release.

Designing nanoMIPs that precisely mimic the natural ligands for BBB receptors is key to employing this highly selective and energy‐dependent process.^[^ ^105^ ^]^ In addition to conventional surface functionalization, a more targeted strategy involves endogenous translocation mechanisms, particularly RMT and carrier‐mediated transport (CMT).^[^ ^312^ ^]^ Although traditionally conceptualized as “barriers,” these pathways operate as finely regulated “gateways” for essential nutrients and biomolecules, presenting valuable opportunities for therapeutic delivery to the CNS. RMT utilizes the interaction between nanoparticle‐bound ligands and specific receptors expressed on brain endothelial cells to facilitate transcytosis. Therefore, nanoMIPs can be engineered with multifunctional surfaces capable of simultaneous binding to distinct ligands or cargos. For example, Figueiredo et al. demonstrated that nanoMIPs functionalized with ApoE not only enhanced receptor targeting but also enabled the codelivery of loaded therapeutic agents, thereby improving BBB penetration via the RMT pathway (**Figure** **8**a).^[^ ^294^ ^]^ Nevertheless, the challenge lies in optimizing the ligand density and presentation on the nanoparticle surface to promote transcytosis while avoiding excessive intracellular retention or lysosomal degradation.^[^ ^313^ ^]^

In addition to ligand selection, the design strategies that can govern RMT efficiency include valency, spacing/orientation, and the biointerface.^[^ ^237^, ^314^ ^]^ The choice of monovalent and moderate‐affinity ligands can favor reducing receptor clustering and endothelial retention. The incorporation of pH‐tunable binding (e.g., histidine‐rich motifs) can minimize the affinity in early endosomes, favoring recycling and transcytotic sorting over lysosomal routing.^[^ ^315^, ^316^ ^]^ Setting the surface density deliberately, keeping a limited number of reactive sites, and backfilling with inert PEG to avoid multivalent patches helps curb unwanted clustering.^[^ ^314^ ^]^ The use of site‐specific conjugation to keep the binding epitope exposed and the selection of short PEG linkers that raise the ligand above the protein corona without causing crosslinking can increase the RMT efficiency.^[^ ^317^ ^]^ Managing the biointerface to keep ligands accessible in serum by maintaining a near‐neutral to slightly negative charge, adopting a brush‐PEG architecture, and using corona‐responsive designs (e.g., preadsorption of dysopsonins) will also be beneficial.^[^ ^318^, ^319^, ^320^, ^321^ ^]^ Therefore, key points include ligand copies per particle, ligand accessibility in serum (e.g., competitive binding), endothelial receptor clustering, and the relationship between apparent permeability and ligand copy number under subsaturating receptor occupancy to avoid downregulation. Together, these measures can improve parenchymal delivery while limiting endothelial sequestration.

NanoMIPs are highly specific, but off‐target binding can still occur in the blood and brain. Key risks are epitope similarity (homologs/isoforms), corona‐mediated masking or representation, charge‐driven AMT/nonspecific endocytosis, and high‐affinity or multivalent displays that cluster receptors and promote endothelial retention. To assess and mitigate these effects, counterscreening against homologs and off‐target cells, testing ligand accessibility and competitive binding in 100% serum, and profiling hard/soft coronas, performing hCMEC/D3 transcytosis with competition/blocking, quantifying receptor clustering, determining apparent permeability to the ligand copy number at subsaturating occupancy, and confirming the biodistribution ± ligand excess in vivo are needed. Mitigations include monovalent, moderate‐affinity formats; controlled ligand density with site‐specific conjugation; near‐neutral to slightly negative *ζ*‐potentials with brush‐PEG; and corona‐responsive surfaces (e.g., dysopsonin preconditioning), as described earlier, considering the valence, density, and corona effects, which address AMT/RMT trade‐offs.

Studies using TfR shuttle antibodies and TfR‐targeted nanoparticles have demonstrated that monovalent ligands with moderate binding affinity facilitate efficient transcytosis across endothelial barriers, whereas bivalent or high‐affinity binding tends to enhance endothelial degradation, thereby reducing delivery to the parenchyma.^[^ ^113^, ^229^, ^230^, ^237^ ^]^ Therefore, to attain optimized RMT, it is necessary to (i) employ monovalent presentation of the imprinted epitope or peptide mimic, (ii) explore the apparent dissociation constant under physiologically relevant serum conditions, (iii) quantify ligand surface density and assess ligand accessibility following protein corona formation, and (iv) conduct dose‐ranging studies, as receptor downregulation is concentration dependent.

Furthermore, since the protein corona can fundamentally alter or even override the intended targeting specificity of nanoMIPs, integrating corona characterization and corona engineering into RMT studies is essential. Comprehensive characterization requires the analysis of hard and soft corona fractions and time‐resolved proteomic profiling to understand corona evolution under physiological conditions. Simultaneously, corona engineering strategies, such as preadsorption of dysopsonins, e.g., clusterin or apolipoproteins, can be employed to modulate corona composition and preserve ligand accessibility. Therefore, incorporating these approaches can improve the translational relevance of in vivo RMT performance for nanoMIPs.^[^ ^233^, ^234^, ^252^, ^307^, ^308^, ^322^ ^]^

For example, despite being a strong TfR‐targeting peptide, THR was unable to mediate the uptake and transcytosis of polymersomes, possibly due to improper presentation on the nanoparticle surface, even with a PEG shield.^[^ ^230^, ^237^, ^323^ ^]^ This highlights the complexity of optimizing ligand presentation for successful RMT. CMT systems, such as GLUT‐1 and large neutral amino acid transporter type 1 (LAT1), transport essential water‐soluble nutrients. While primarily used for small molecules, prodrug strategies involving MIPs could be designed to exploit these transporters, although this area is less explored for polymeric nanoparticles.^[^ ^324^, ^325^ ^]^ A specific example of LAT1‐targeted nanoparticles is phenylalanine‐functionalized nanoparticles, which deliver antisense oligonucleotides across the BBB via LAT1; however, this approach is limited in scope and not polymer‐based in the mainstream delivery sense.^[^ ^326^ ^]^ Thus, harnessing CMT systems via MIP‐enabled polymeric nanoparticles represents a potentially fruitful yet developing research frontier.

Ligand choice for nanocarrier surface modification critically shapes RMT performance across experimental models. Angiopep‐2 (a ligand of LRP1) often outperforms transferrin derivatives in both in vitro transcytosis and in situ rodent perfusion. For example, studies have reported greater transcytosis capacity and noticeably greater brain (parenchymal) volume distribution for angiopep‐2 than transferrin in hCMEC/D3 and in situ mouse perfusion assays.^[^ ^327^, ^328^, ^329^, ^330^ ^]^ ApoE‐functionalization yields moderate, reproducible boosts in hCMEC/D3 and coculture models (≈1.5–1.9 times higher permeability/transport), consistent with our discussion above, but in vivo brain drug measurements can underreflect this gain when part of the cargo remains complexed to the imprinted binding sites during transit.^[^ ^294^, ^331^ ^]^ Transferrin/TfR strategies are potent yet constrained by ligand‐specific liabilities: endogenous holo‐transferrin competes for a limited number of TfR1 sites, and high‐affinity or bivalent TfR binders can be sequestered and degraded in the endothelium, producing the characteristic bell‐shaped “exposure‐versus‐affinity” relationship and concentration‐dependent receptor downregulation (brain “sink” saturation).^[^ ^230^, ^237^, ^332^, ^333^ ^]^ Angiopep‐2 is not exempt from trade‐offs. LRP1 is also expressed in the liver and other peripheral tissues, and angiopep‐2 conjugates (e.g., ANG1005) show substantial hepatic and reticuloendothelial uptake alongside robust brain delivery.^[^ ^329^, ^330^, ^334^ ^]^ Therefore, practical selection hinges on disease biology for brain tumors and metastases where LRP1 is frequently elevated on the tumor endothelium and tumor cells. Angiopep‐2 shuttles have shown strong preclinical efficacy and clinical translation (ANG1005).^[^ ^335^ ^]^ For neurodegeneration, where widespread neuronal and endothelial TfR1 persists and neuronal uptake is desired, optimized low‐affinity/monovalent TfR formats avoid endothelial trapping and can deliver biologics broadly to the parenchyma.^[^ ^230^, ^237^, ^333^ ^]^ ApoE‐decorated nanocarriers remain attractive adjuncts for neurodegeneration when a modest, isoform‐agonistic boost to BBB flux is sufficient and off‐target uptake must be minimized.

### 5.2 Cellular Hitchhiking

Cellular hitchhiking has emerged as a novel and promising strategy for delivering nanocarriers across the BBB by exploiting the native migratory functions of circulating immune cells.^[^ ^309^ ^]^ Neutrophils, a type of white blood cell capable of traversing endothelial barriers in response to inflammation, serve as attractive Trojan‐horse vehicles for this purpose. Recent work has demonstrated that nanoparticles can be designed to bind to neutrophils, leveraging their integrins to facilitate binding and subsequent transmigration into the central nervous system.^[^ ^336^, ^337^ ^]^ This approach exploits the inherent migratory capabilities of cells, potentially leading to enhanced and targeted delivery to pathological regions of the brain, such as those affected by ischemic stroke.^[^ ^338^, ^339^, ^340^ ^]^ In glioblastoma therapy, neutrophil‐mediated delivery systems have shown promising preclinical results. For example, neutrophils loaded *ex vivo* with drug‐containing nanoparticles, such as paclitaxel‐loaded cationic liposomes (PTX‐CL/NEs), successfully migrated to residual tumor sites, releasing their therapeutic payload and preventing tumor recurrence in mouse models.^[^ ^341^ ^]^ These approaches have the advantages of localized treatment while minimizing systemic exposure and side effects. In addition to cellular hitchhiking, peptide‐based hitchhiking strategies are also being explored. Peptide‐functionalized nanoparticles, known as “peptide hitchhiking”, have shown potential for enhancing BBB penetration and glioblastoma targeting, facilitated by the ease of peptide synthesis and high selectivity for brain or tumor markers.^[^ ^342^ ^]^ This strategy is still in its nascent stages but holds significant promise for noninvasive and highly targeted delivery of therapeutics across the BBB, particularly for conditions involving inflammation where immune cell infiltration is relevant. While still in early stages, these strategies highlight the potential of peptide‐decorated nanoMIPs in noninvasive, highly targeted CNS delivery, particularly in pathologies driven by inflammation, where immune cell infiltration plays a key role. A comprehensive summary of the strategies widely used to improve targeted transport across the BBB is provided in **Table** **4**.

**Table 4:** Summary of strategies to enhance targeted therapeutic transport across the BBB, highlighting underlying mechanisms and representative examples.

Strategy	Mechanism	Examples	Ref.
Neutrophil hitchhiking	Nanoparticles bind neutrophils via integrins and cross BBB into diseased brain regions	Feasibility in CNS via *α*v*β*3 integrin‐mediated uptake.	[336]
Inflammation‐enabled targeting	Neutrophils respond to inflammation, aiding NP delivery to ischemic or inflamed brain areas	Strategies for CNS nanoparticle delivery using neutrophil migration.	[340]
Glioblastoma targeting	Neutrophil‐loaded liposomal therapeutics reducing tumor recurrence in mice	Paclitaxel‐loaded neutrophil delivery prevented GBM recurrence.	[341]
Peptide hitchhiking	Peptide ligands enhance nanoparticle targeting and brain penetration.	Peptide‐functionalized NP strategies for glioblastoma	[342]

Although neutrophil‐mediated nanoparticle hitchhiking has demonstrated convincing proof‐of‐concept efficacy in glioma and other inflammatory models, quantitative studies reveal that precise control of neutrophil loading remains challenging. *Ex vivo* experiments have shown that human neutrophil uptake occurs rapidly (within ≈15–120 min) but is strongly influenced by serum proteins as well as by particle chemistry and size. Notably, preadsorption of albumin can increase the uptake of certain nanoparticle formulations.^[^ ^343^, ^344^ ^]^ In vivo, however, only a small fraction of circulating neutrophils, typically ≤ 0.5% in tumor models, interact with administered nanoparticles, highlighting the difficulty of achieving consistent and efficient loading through systemic administration alone.^[^ ^345^ ^]^ Consequently, hitchhiking studies should also investigate quantitative loading metrics, such as the number of particles per cell, along with viability and activation markers, e.g., CD62L shedding, and functional migration capacity following nanoparticle loading.

Neutrophils can aggravate CNS injury through NETosis and the release of neutrophil serine proteases, processes that exacerbate inflammation in stroke and other neuroinflammatory disorders.^[^ ^346^, ^347^, ^348^ ^]^ Consequently, the use of neutrophil‐based delivery systems may involve context‐dependent risks of amplifying neuroinflammation within diseased brain tissue. To mitigate these risks, several strategies have been proposed. These include minimizing ex vivo activation during cell handling, employing membrane‐coated nanocarriers or neutrophil‐derived vesicles instead of live neutrophils, and, where appropriate, incorporating NET‐modulating or anti‐inflammatory payloads to counteract excessive immune activation.

Neutrophils can be loaded ex vivo through brief incubation with nanoMIPs that carry low‐valence ligands for surface markers (e.g., CD11b, PSGL‐1), followed by reinfusion of autologous cells.^[^ ^349^, ^350^ ^]^ In vivo, systemic administration of nanoMIPs bearing neutrophil‐binding motifs enables transient, reversible adsorption to circulate neutrophils without cell involvement.^[^ ^351^, ^352^, ^353^ ^]^ In both approaches, the ligand density and particle concentration should be kept low to avoid receptor clustering and activation. Several studies have reported that viability, chemotaxis, phagocytosis, and oxidative bursts remain within baseline ranges when loading stays within this window and that trafficking to inflamed tissue is preserved.^[^ ^354^ ^]^ High loading or strongly agonistic ligands can reduce chemotaxis; therefore, hitchhiking is best treated as surface adsorption rather than internalization. A standard immunotoxicity panel (viability, ROS, cytokines, and migration) during dose determination is recommended to confirm that core immune functions are not significantly impaired.^[^ ^355^ ^]^

### 5.3 Controlled Drug Release Triggered by the Brain Microenvironment

Finally, developing responsive and controlled drug release systems that are triggered by the unique microenvironment of the brain as well as external stimuli offers an intelligent approach to optimize therapeutic delivery (Figure 8b). The brain microenvironment can differ significantly between healthy and diseased states in terms of pH, temperature, enzyme concentrations, and the presence of specific biomarkers or oxidative stress. Stimuli‐responsive MIPs (SR‑MIPs), also known as “smart” or “intelligent” drug delivery systems, are engineered to release their payload in response to such localized cues.^[^ ^356^, ^357^, ^358^ ^]^ By incorporating stimuli‐responsive comonomers into the MIP matrix, a polymer's binding affinity or permeability can be modulated in situ, enabling triggered release. For example, pH‑responsive systems are well studied: diseased tissues such as tumors or ischemic brain regions often present a lower pH than healthy tissues do, and pH‑responsive polymeric carriers can undergo conformational changes or degradation to release their drug cargo preferentially in acidic environments. Recently, Liu et al. reported the synthesis of MIPs via atom transfer radical polymerization (ATRP) via the use of 4‐amino‐4′‐methacrylamide azobenzene and *β*‐cyclodextrin as bifunctional monomers and N,N’‐bis(acryloyl)cystamine and ethylene glycol dimethacrylate as cross‐linkers, while andrographolide (ADR) served as the template.^[^ ^359^ ^]^ This MIP exhibited excellent stability and reproducibility, and its in vitro release behavior demonstrated that ADR‐MMIP could achieve controlled ADR release through triple pH/redox/light‐responsive bonds. The cumulative drug release rate was found to be ≈91.5% when all three stimuli were used simultaneously. In the enzyme‑responsive context, overexpressed enzymes in diseased brain tissue (e.g., matrix metalloproteinases or other proteases) can cleave localized linkers in the MIP, triggering site‐specific drug release.^[^ ^360^ ^]^ Likewise, redox‑responsive systems, which exploit elevated levels of reactive oxygen species (ROS) or intracellular reducing agents such as glutathione in neuroinflammation or stroke, can employ redox‑cleavable bonds that respond to the oxidative state of the local microenvironment.^[^ ^361^, ^362^, ^363^ ^]^ Finally, biomarker‑responsive MIPs, which are designed to recognize and respond to a specific disease biomarker, offer the potential for highly targeted and localized drug release; however, while such systems are less common in the brain‑delivery field, they represent a frontier in smart drug delivery technology.

In most settings, pathological cues in the brain are modest and heterogeneous. On their own, they rarely drive drug release to a therapeutically adequate concentration across the whole lesion. pH shifts are mild; enzyme expression varies by region and time; and redox states fluctuate. These features limit the release rates and total payload mobilized. Diffusion distances, protein‒corona shielding, and endosomal sequestration can further blunt effective exposure. For this reason, framing stimuli responsiveness is considered a sharpening tool rather than the sole dosing engine. In practice, pairing triggers with BBB‐transport strategies, moderate‐affinity binding to avoid endothelial trapping, and preloading sufficient cargo are highly important. Dual‐ or multitrigger designs can widen the release window while keeping off‐target leakage low (Figure 8b). Together, these measures improve the chance of reaching therapeutic levels despite the kinetic and payload limits of stimuli‐responsive release.^[^ ^364^, ^365^, ^366^, ^367^ ^]^

This controlled and sustained release capability represents a significant advantage: it can prolong the therapeutic effect, reduce the dosing frequency, and minimize systemic toxicity by ensuring that the drug is released predominantly at the pathological site.^[^ ^368^ ^]^ Precise control over drug release kinetics, combined with targeted delivery, maximizes the therapeutic index and improves patient outcomes. Thus, a combinatorial approach that integrates advanced strategies, such as surface modification with targeting ligands, exploitation of endogenous transport mechanisms, cellular hitchhiking, and stimuli‑responsive release systems, is essential for enhancing the brain delivery of nanoMIPs and unlocking their full potential in treating complex neurological diseases.

### 5.4 Delivery of Antiviral Agents, Chemotherapeutics, Neuroprotectants, and Other CNS Drugs

nanoMIPs hold immense potential for the targeted delivery of a wide array of therapeutic agents, including antiviral agents, chemotherapeutics, neuroprotectants, and other CNS‐specific drugs, to the central nervous system. By virtue of their engineered specificity, nanoMIPs can overcome these limitations.^[^ ^250^ ^]^

For antiviral applications, nanoMIPs could be tailored to recognize and bind viral proteins or components involved in CNS infections, such as HIV‑1 in neuro‑AIDS, thereby delivering antiviral drugs directly to infected cells or viral reservoirs within the brain and reducing systemic exposure while improving treatment efficacy. Although specific demonstrations of this concept remain scarce, the general principle is supported by the broader field of targeted MIP‐based delivery.^[^ ^49^ ^]^

In the field of chemotherapeutics, brain tumors, particularly aggressive forms such as glioblastoma, are extremely difficult to treat because of the restrictive nature of the BBB and rapid and infiltrative growth of tumors. NanoMIPs can be imprinted with specific tumor biomarkers or drug targets, allowing for the precise delivery of anticancer drugs. This targeted delivery can increase the accumulation of chemotherapeutics within tumor cells while minimizing exposure to healthy brain tissue, thereby improving the therapeutic index and reducing severe systemic toxicity. For example, magnetic MIPs have been developed as intelligent drug delivery systems for anticancer agents such as 5‐fluorouracil, demonstrating the potential of this approach.^[^ ^369^ ^]^ Similarly, biodegradable nanoMIPs have been reported by Yoosefi et al. for the delivery of methotrexate, an anticancer drug, highlighting the development of promising carriers for cancer therapy.^[^ ^370^ ^]^

Neuroprotectants are crucial for mitigating neuronal damage in conditions such as stroke, traumatic brain injury, or neurodegenerative diseases. MIPs can be engineered to deliver these agents to specific neuronal populations or glial cells that are most vulnerable or contribute to pathology. For example, poly(butyl cyanoacrylate) nanoparticles have demonstrated the ability to deliver large molecules such as horseradish peroxidase (HRP) and enhanced green fluorescent protein (EGFP) to injured brain sites in rats with TBI, where they can evade the BBB.^[^ ^371^ ^]^ Although not explicitly MIPs, this finding illustrates the potential for nanocarriers to deliver neuroprotective agents to specific brain regions under pathological conditions.

In addition to these categories, MIPs can also be tailored for the delivery of a wide range of other CNS drugs, including those for epilepsy, psychiatric disorders, or chronic pain, where sustained and targeted delivery can significantly improve therapeutic outcomes and reduce side effects associated with systemic administration.^[^ ^372^ ^]^ The ability of MIPs to selectively recognize and prolong the release of drugs, even under physiological buffer conditions, makes them highly promising candidates for such applications.

### 5.5 Benchmarks for an “Ideal” Brain‐Targeting nanoMIP

In summary, imprinting factor values of ≥5 in buffer and ≥3 in protein‐containing media represent practical targets, with ≥10 desirable when attainable, as this level often reflects stronger selectivity over nonimprinted particles.^[^ ^373^, ^374^, ^375^ ^]^ For drug‐loading capacity (DLC%), ≥10 wt% may be considered a reasonable baseline for polymeric nanoparticles, whereas 15–30 wt% is often preferable when drug‒polymer compatibility permits.^[^ ^376^, ^377^, ^378^ ^]^ However, the emerging high‐loading strategies could exceed these ranges. For in vitro BBB permeability of small‐molecule cargo released from nanoMIPs, apparent permeability ≳ 2–4 × 10^−6^ cm·s^−1^ in high‐integrity human BBB models appears to be a reasonable indicator of brain entry and is broadly consistent with parallel artificial membrane permeability assay for the BBB (PAMPA‐BBB) “BBB+” cutoffs.^[^ ^379^ ^]^ Models with documented barrier tightness (e.g., iPSC‐BMECs with transepithelial/transendothelial electrical resistance (TEER) > 1000 Ω·cm^2^) may be preferable.^[^ ^380^, ^381^ ^]^ Inclusion of internal controls, such as lucifer yellow (a paracellular marker), together with caffeine or propranolol, facilitating calibration and comparison, are recommended.^[^ ^382^ ^]^ Because hCMEC/D3 monolayers typically exhibit a lower TEER and limited tightness, the apparent permeability thresholds obtained in this system should be interpreted with appropriate caution. Overall, these ranges reflect commonly reported performances for MIPs and polymeric nanocarriers and established BBB benchmarks. They are intended as comparability targets rather than strict pass/fail criteria and are most informative when accompanied by assay conditions (TEER, shear, donor characteristics, and controls).

## 6 Pharmacokinetics, Safety, and Biocompatibility of nanoMIPs

The comprehensive evaluation of nanoMIPs for brain disease therapy necessitates rigorous assessment of their pharmacokinetic profiles, safety, and biocompatibility. These evaluations are critical for validating their performance, ensuring their efficacy, and ultimately facilitating their translation from laboratory research to clinical application.

### 6.1 Imprinting Factors and Drug Loading Capacity

“Imprinting factors” (IFs), also known as “relative selectivity coefficients”, are quantitative measures that directly reflect the effectiveness of the molecular imprinting process. They compared the binding affinity or adsorption capacity of the MIP for the target molecule relative to that of an NIP lacking the specific recognition sites.

**Figure 9:**
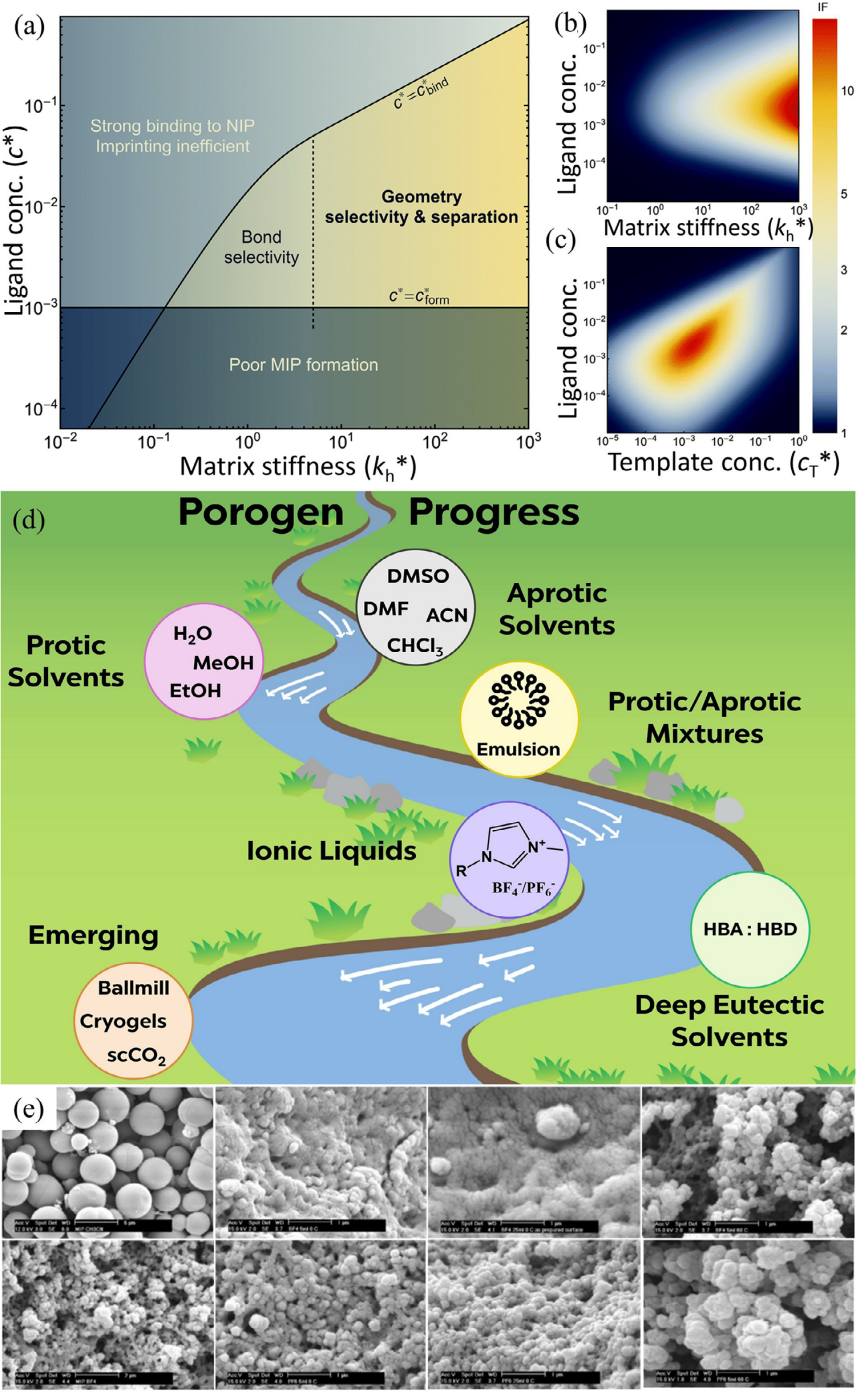
Design principles of molecularly imprinted polymers (MIPs). a) Schematic phase diagram illustrating MIP efficiency and summarizing key design considerations. The optimal ligand concentration for efficient imprinting lies between two critical thresholds. The dashed line demarcates regions dominated by bond selectivity versus geometrical recognition. Reproduced with permission.^[^ ^270^ ^]^ Copyright 2016, Author(s). b) Imprinting factor for divalent templates as a function of template concentration and 𝑐∗ at a stoichiometric ligand‒receptor ratio. Reproduced with permission.^[^ ^270^ ^]^ Copyright 2016, Author(s). c) Imprinting factor as a function of template concentration and 𝑐∗ at fixed matrix stiffness, assuming equal binding strengths during both the imprinting and binding stages. Reproduced with permission.^[^ ^270^ ^]^ Copyright 2016, Author(s). d) Different porogenic solvents, ranging from aprotic solvents to emerging solvents, have been used over time in MIP synthesis. Reproduced with permission.^[^ ^387^ ^]^ Copyright 2025, Author(s). e) SEM images illustrating the effects of ionic liquid‐based porogens on the MIP morphology and cavity fidelity. Varying porogen volumes at different polymerization temperatures reveal that ionic liquid‐based porogens play a critical role in determining the morphology, particle size, pore architecture, and cavity fidelity of molecularly imprinted polymers. Reproduced with permission.^[^ ^388^ ^]^ Copyright 2007, CSIRO.

Maximizing the IF in MIP synthesis depends critically on optimizing key parameters to maximize recognition site fidelity and density. First, the template‐to‐monomer ratio plays a decisive role: a lower template‐to‐monomer ratio often enhances binding efficiency by increasing the relative number of homogeneous recognition sites, whereas excessive monomers can reduce rigidity and selectivity, lowering the IF.^[^ ^270^, ^383^, ^384^ ^]^ Template:monomer:cross‐linker ratios have been shown to profoundly affect performance; for example, a ratio of 1:6:20 yielded an IF of ≈3.94, which is much higher than other tested ratios. This highlights the need to balance interaction opportunities with structural integrity.^[^ ^385^ ^]^ Cross‐linker density itself governs physical properties and binding site preservation; overly dense networks may obstruct access to internal cavities, whereas excessively sparse cross‐linking compromises binding site fidelity and polymer robustness.^[^ ^287^ ^]^ A moderate cross‐link density is therefore essential to maintain both capacity and selectivity. Finally, computational modeling, including artificial neural networks and quantum descriptors, has demonstrated strong predictive power for the IF based on template‐monomer and mobile‐phase characteristics, enabling more efficient optimization before laboratory synthesis.^[^ ^386^ ^]^ Curk et al. developed a model that shows that optimal molecular imprinting requires a near‐stoichiometric ratio between ligands and templates, ensuring efficient cavity formation and selective analyte rebinding (e.g., a 1:3 ratio for trivalent templates) (**Figure** **9**a–c).^[^ ^270^ ^]^ The initial ligand concentration should be comparable to the bond dissociation constant to balance cavity quality with binding specificity. Furthermore, matrix stiffness critically influences selectivity: stiffer matrices increase geometrical recognition but may hinder analyte access and slow binding kinetics. While grinding stiff gels can expose more cavities, it partially damages ligand sites, reducing imprinting quality. Therefore, an application‐specific tradeoff between matrix rigidity, accessibility, and binding efficiency is essential for optimizing MIP performance. Solvent or porogen selection plays a crucial role in shaping the cavity fidelity, pore architecture, and overall binding performance of MIPs.^[^ ^387^ ^]^ Although aprotic solvents have traditionally dominated MIP synthesis, recent advancements have introduced a broader range of porogens, leading to significant enhancements in MIP efficiency and selectivity (Figure 9d).^[^ ^387^ ^]^ Booker et al. carried out a systematic study using different volumes of ionic liquids as green solvents at different temperatures and demonstrated that porogens can govern the morphology, pore architecture, cavity fidelity, and binding performance of MIPs by modulating polymer network formation during synthesis (Figure 9e).^[^ ^388^ ^]^ An IF greater than unity indicates successful imprinting and superior selectivity of the MIP toward its template. As mentioned earlier, studies have shown that nanoMIPs can detect target proteins with several times greater efficiency than NIP nanoparticles can, indicating a significant imprinting effect. High IFs are important for targeted drug delivery, ensuring that the therapeutic payload preferentially binds to the intended biological target within the brain, minimizing off‐target effects.

The drug loading capacity (DLC) of nanoMIPs is a crucial metric, indicating the amount of therapeutic agent that can be effectively incorporated into the polymer matrix. A high DLC is desirable, as it allows for the delivery of therapeutically relevant drug concentrations with fewer nanoparticles, potentially reducing the overall material burden in vivo. DLC is influenced by factors such as the choice of functional monomers, the cross‐linker density, the polymerization method, and the nature of the drug template itself. MIPs, by design, often demonstrate high drug loading capacity owing to the specific cavities created for the template molecule.

### 6.2 Release Kinetics and Sustained Drug Delivery Profiles

Sustained drug delivery profiles from MIPs are achieved through various design considerations. The specific binding sites within the MIP matrix act as reservoirs, retaining the drug and releasing it gradually over time.^[^ ^372^ ^]^ The rate of release can be influenced by factors such as the strength of the interaction between the drug and the MIP's binding sites, the cross‐linking density of the polymer, the particle size, and the environmental conditions (e.g., pH, temperature, and enzymatic activity). For example, dipyridamole‐imprinted polymers prepared by precipitation polymerization showed very slow release rates in various physiological solutions (e.g., phosphate buffer and HCl) and were able to prolong drug release for more than two days.^[^ ^163^ ^]^ Similarly, nicotinamide‐based MIP microspheres demonstrated selective rebinding and prolonged release compared with non‐imprinted systems.^[^ ^389^ ^]^ The ability to modulate release in response to specific stimuli (stimuli‐responsive systems) offers an advanced level of control, allowing for “on‐demand” drug release at the pathological site. This can lead to more effective therapy, reduced concentration frequency, and improved patient compliance.

### 6.3 In Vitro and In Vivo Biocompatibility with Toxicity Data

Biocompatibility and toxicity assessments are fundamental to ensure the safety of MIP nanocarriers for clinical use. In vitro studies typically involve assessing cytotoxicity, cellular uptake, and barrier integrity via models of the BBB, such as brain endothelial cell lines (e.g., hCMEC/D3 cells) or more complex in vitro BBB‐on‐a‐chip models.^[^ ^390^ ^]^ These models allow researchers to investigate how nanoMIPs interact with brain endothelial cells, their internalization efficiency, and any potential detrimental effects on cell viability or barrier function. For example, polymeric nanoparticles have shown satisfactory cell compatibility and permeation into spheroid cores in 3D BBB models.^[^ ^391^ ^]^ Studies have also confirmed that certain polymeric nanoparticles can be internalized by brain endothelial cells with acceptable toxicity profiles at relevant concentrations.^[^ ^392^ ^]^ In vivo investigations of solid‐phase synthesized nanoMIPs by Kassem et al. have presented studies on their biodistribution, clearance, and cytotoxicity in rat models.^[^ ^265^ ^]^ The initial results indicate extended circulatory persistence and targeted tissue accumulation without pronounced toxicity; however, comprehensive long‐term safety data are lacking.

### 6.4 Immunogenicity and Long‐term Safety Considerations

In addition to immediate toxicity, the immunogenicity of nanoMIPs is a critical long‐term safety consideration. As synthetic materials, MIPs can potentially trigger an immune response, leading to rapid clearance, inflammation, or other adverse effects. Strategies such as PEGylation are often employed to create surface‐modified nanoparticles that evade immune detection, thereby reducing immunogenicity and prolonging circulation. The inherent biocompatibility of some polymer components, such as polypyrrole, which does not irritate the mammalian immune system, also offers promising prospects.

Long‐term safety considerations also involve assessing potential chronic toxicity, accumulation in nontarget organs, and the biodegradability of the polymer matrix. While MIPs have demonstrated robust durability and stability, their degradation products and long‐term fate in vivo need to be thoroughly investigated. The ideal nanoMIPs would be fully biodegradable into nontoxic components after fulfilling their therapeutic role. Continuous monitoring and rigorous regulatory evaluations are crucial steps toward the clinical approval of MIP nanocarriers for brain disease therapy.

Therefore, a robust evaluation framework encompassing DLC, IFs, precise release kinetics, and comprehensive in vitro and in vivo safety assessments, including immunogenicity and long‐term effects, is essential to realize the full therapeutic potential of nanoMIPs for the treatment of brain diseases.

## 7 Current Challenges and Limitations

Despite the immense promise of nanoMIPs for crossing the BBB and treating brain diseases, their widespread clinical translation faces significant challenges and limitations. These issues are due to synthetic hurdles, scalability to regulatory barriers, and in vivo tracking difficulties.

### 7.1 Solubility and Stability of Templates and Monomers During Synthesis

One of the foundational challenges in MIP synthesis, particularly for biological templates, is related to the solubility and stability of the templates and monomers during the polymerization process. Many biological molecules, such as proteins and peptides, are highly sensitive to the organic solvents often used in conventional imprinting methods. Their denaturation or degradation during the prepolymerization step can lead to nonspecific binding sites or loss of template integrity, reducing the selectivity and affinity of the final MIP. For example, while proteins are crucial disease biomarkers, their complex structures and varying physicochemical characteristics, such as hydrophilicity, hydrophobicity, and charge, make them challenging templates to mimic. The need for water‐compatible MIPs and the development of alternative synthesis methods, such as Pickering emulsion polymerization or coprecipitation, have emerged to overcome the limitations of organic solvent usage. Furthermore, the interactions between monomers and comonomers and their stability during the polymerization process are important. Molecular dynamics simulations can reveal low interaction energies between certain monomers, indicating the necessity of a polymerization agent to ensure cross‐linked stable polymer formation. The presence of unreacted monomers or byproducts can also increase concerns about biocompatibility and necessitate extensive purification steps.

### 7.2 Scalability and Reproducibility of nanoMIPs

The scalability and reproducibility of nanoMIP nanocarrier fabrication present considerable hurdles for industrial application. Conventional bulk synthesis methods are often time‐consuming, e.g., a day or more, labor‐intensive (multiple steps), and require sophisticated equipment, which impedes high‐throughput production. Moreover, these batch processes are prone to variations in particle size, defects, and stability across different batches, severely impacting reproducibility. Minute perturbations in experimental conditions can hinder polymerization and growth, leading to low yields and the production of polydisperse particles.

The transition from lab‐scale synthesis to large‐scale manufacturing of nanoMIPs faces specific bottlenecks. Increased volumes of reagents and dispersion volumes necessitate precise control for uniform mixing, which is challenging to maintain consistently. This variability hinders their full phase of clinical translation.

Microfluidic technologies offer promising solutions to these challenges, enabling in situ and continuous synthesis of nanoMIPs with improved reproducibility and control. Microreactors can produce nanoparticles in a shorter time frame, e.g., five minutes to half an hour, with higher yields and better monodispersity, reducing the need for multiple centrifugation steps for size separation. However, challenges remain with microfluidic systems, such as potential clogging of microchannels owing to polymer smears, ensuring simultaneous introduction of reagents with different flow rates, and the inherent miniaturized nature that limits the total volume processed in a single device. While parallelization via the use of multiple microreactors simultaneously can increase throughput, further advancements are needed for truly large‐scale, cost‐effective, and highly reproducible production.

### 7.3 Limited Clinical Translation and Regulatory Barriers

Despite robust scientific advancements, the limited clinical translation and associated regulatory barriers remain another major challenge for nanoMIPs. Very few, if any, MIP‐based drug delivery systems have reached advanced clinical trial stages, especially for brain applications.^[^ ^393^, ^394^ ^]^ This lag is attributable to several factors, such as the knowledge gaps in in vivo studies. It is challenging to define the in vivo fate of nanoMIPs precisely, particularly within the brain, which is a stringent regulatory requirement. This includes understanding their biodistribution, degradation pathways, and long‐term retention or clearance in various organs. The other factor is their complex formulation and sophisticated characterization, which are required for nanocarriers and make regulatory approval cumbersome. Ensuring batch‐to‐batch consistency in properties such as size, surface chemistry, drug loading, and release kinetics is crucial for pharmaceutical products.^[^ ^393^ ^]^ In addition, while in vitro studies show promise, extensive in vivo preclinical data on long‐term toxicity, immunogenicity, and potential systemic side effects are needed before human trials. The potential for nonspecific binding and accumulation in peripheral organs, especially for nonspecifically targeted carriers, needs thorough investigation.

The development and manufacturing costs for novel nanocarrier systems can be high, and their perceived cost‒benefit ratio in comparison with existing therapies needs to be attractive for regulatory bodies and pharmaceutical companies. The field of brain‐targeted drug delivery has also seen competition from other platforms, such as targeted liposomes and monoclonal antibody‒drug conjugates, which may have more established regulatory pathways or a longer clinical history.

NanoMIPs must overcome several practical hurdles to compete with antibody carriers. First, pharmacokinetic definition and scaling: establish human‐relevant ADME with population variability, including reticuloendothelial clearance and metabolite identification. Second, surface/identity control involves locking down batch‐to‐batch ligand density, orientation, and corona profiles under pharmaceutically realistic matrices. Third, CMC robustness demonstrates high‐throughput, in‐process controls for imprint uniformity, residuals (monomers/initiators), and degradants. Fourth, immuno‐ and hemocompatibility should be assessed to assess complement activation, thrombogenicity, and antipolymer responses after repeated dosing. The fifth criterion is assay translatability: qualify BBB models and transcytosis assays against in vivo benchmarks and define decision criteria for go/no‐go. Finally, regulatory mapping prespecifies critical quality attributes (CQAs) for the imprinted shell and surface display and aligns release tests with these CQAs to support a predictable clinical path. Together, these steps narrow the current advantage that antibodies hold from well‐defined PKs and established pathways. Overcoming all these hurdles will require standardized testing protocols, robust manufacturing processes, and comprehensive long‐term safety data.

#### 7.3.1 Regulatory Pathways: nanoMIPs, Liposomes, and Polymeric Nanoparticles

Compared with nanoMIPs, liposomal products are supported by well‐designed product‐specific guidelines. In the United States, the Food and Drug Administration's (FDA) “Liposome Drug Products” guidelines (April 2018) explicitly cover Chemistry‐Manufacturing‐Controls (CMC), human pharmacokinetics/bioavailability, and labeling and identify core CQAs, including vesicle/particle size and distribution, morphology, net surface charge, encapsulation and drug loading efficiency, in vitro release, leakage during shelf‐life, and response to stimuli, along with prospects for process control, specifications, and stability studies.^[^ ^395^ ^]^ Similarly, the European Medicines Agency (EMA) has set quality requirements for intravenous liposomal products. These include characterization of morphology, particle size, and size distribution, and the degree of aggregation must be assessed. The fraction of free versus entrapped drug should be quantified, and the residual fragments from degrading lipids or drugs should also be measured. EMA also recommends that in vitro release testing be performed in clinically relevant media via reliable methods. It also has nonclinical and clinical comparability, as liposomal formulations can reduce clearance and volume of distribution along with prolonged half‐life, which must be addressed with comparative pharmacokinetics and distribution data.^[^ ^396^ ^]^ On the other hand, for polymeric nanocarriers, guidelines are more specific to their classes. The joint Ministry of Health, Labor and Welfare/EMA guidelines on block copolymer micelle products emphasize intravenous use and outlines pharmaceutical development controls, first‐in‐human planning, and nonclinical/clinical pharmacokinetics, including collection of the peak plasma concentration of a drug after dosing, half‐life, overall drug exposure for both total and unencapsulated drugs, frequent early sampling, and organ/tumor distribution time profiles.^[^ ^397^ ^]^ In 2022, the FDA issued guidance on drugs and biologics that contain nanomaterials. It uses a risk‐based approach. Researchers should fully characterize nanomaterials and link their properties to product quality, safety, and efficacy. They should also assess in vivo release, the potential for immune reactions, and how manufacturing changes could affect critical quality attributes (CQAs).^[^ ^398^ ^]^ Therefore, nanoMIPs need to be evaluated within these frameworks. However, these nanocarriers may require additional scrutiny of template removal and residue, cross‐link density and biodegradability, binding site heterogeneity, and the in vivo fate of highly cross‐linked matrices.

#### 7.3.2 Long‐Term Biocompatibility and Chronic Immunogenicity of Highly Crosslinked nanoMIPs

Highly cross‐linked matrices, e.g., EGDMA‐rich networks, degrade slowly and can persist in tissues. Therefore, evaluation must extend beyond acute safety to chronic immunologic and fate endpoints. Complement activation and infusion reactions should be monitored with in vitro hemocompatibility assays and CH50/C3a/C5a/sC5b‐9 panels, with signals confirmed in appropriate in vivo models. In patients receiving liposomal amphotericin B, rapid postinfusion increases in C3a and sC5b‐9 within 5–30 minutes link complement activation‐related pseudoallergy (CARPA) to infusion reactions.^[^ ^399^, ^400^ ^]^ Antipolymer immune responses must also be considered. Preexisting anti‐PEG antibodies are common in the general population, ≈56–72% overall. In a 300‐donor study, ≈65% had such antibodies. These antibodies have been linked to hypersensitivity reactions and accelerated blood clearance. Therefore, baseline and on‐treatment IgM/IgG screening is recommended. In addition, non‐PEG surface options (e.g., zwitterionic coatings) should be considered.^[^ ^401^, ^402^, ^403^ ^]^ Degradation and tissue persistence should be characterized by mass balance and metabolite identification (including polymer fragments), late‐time histopathology, and quantitative biodistribution/clearance, for example, by radiolabeled quantitative whole‐body autoradiography (QWBA).^[^ ^404^ ^]^ Accordingly, investigations should include an extended repeated‐dose toxicity study appropriate for the intended dosing duration, typically 3 months, as recommended by the International Council for Harmonization [ICH].^[^ ^405^ ^]^ Hemocompatibility/complement testing, targeted immunogenicity assays (anti‐PEG and anti‐matrix as applicable), quantitative whole‐body biodistribution and clearance, degradant/impurity specifications linked to CQAs, and repeat‐dose pharmacokinetics to detect accelerated clearance are needed. In summary, long‐term safety testing should focus on complement activation and infusion reactions, antipolymer immunity, and degradation‐driven tissue persistence.^[^ ^400^, ^406^, ^407^ ^]^

#### 7.3.3 A Practical Roadmap for First‐in‐Human nanoMIP Studies

Therefore, to prepare a practical roadmap for first‐in‐human nanoMIP studies, the following steps are recommended. The guidelines should i) define nanoMIP‐specific CQAs, for example, imprinting‐factor distribution, residual template and cross‐linker levels, and binding‐site accessibility after protein‒corona formation; ii) establish a justified design space and validate stability‐indicating analytical methods; iii) structure nonclinical and early clinical plans using FDA liposome guidance and EMA liposome/micelle reflections, adding nanoMIP‐specific assays such as complement‐activation panels and degrading fragment profiling wherever necessary; iv) predefine an immunosafety plan and on‐treatment anti‐PEG and antipolymer testing, early‐infusion complement monitoring and prespecified stopping rules; and v) document in vivo fate early through standardized biodistribution, clearance, and degradation studies that address two questions: where does the material go, and for how long?

### 7.4 Challenges in In Vivo Imaging and Tracking

Effective in vivo imaging and tracking of MIP nanocarriers within the brain pose significant challenges, yet this capability is crucial for understanding their biodistribution, target engagement, and therapeutic efficacy in real time.

Real‐time visualization of nanoparticles in vivo often requires the attachment of fluorescent dyes or other tracing molecules, which can complicate the synthesis process and potentially alter the nanoparticle's properties or behavior. While some novel fluorescent polymers have been developed for this purpose, comprehensive, high‐resolution, noninvasive real‐time tracking within the complex brain environment remains difficult. Conventional optical imaging techniques often have limited penetration depth in biological tissues, making tracking nanoparticles deep within the brain difficult. Magnetic resonance imaging (MRI) offers better penetration but requires the incorporation of suitable contrast agents into the nanoMIPs. Furthermore, precisely distinguishing between specific accumulation at the target site and nonspecific retention in surrounding healthy brain tissue or other organs is a complex imaging challenge. Monitoring the long‐term fate, degradation, and clearance of nanoparticles in vivo is essential for safety assessments but is technologically demanding. Advances in multimodal imaging, such as combining optical and magnetic resonance imaging and the integration of theranostic platforms (combining therapeutics and diagnostics), could provide solutions by allowing real‐time monitoring of drug delivery and therapeutic response. However, further innovations in imaging technologies and the design of easily traceable nanoMIPs are needed to fully address these limitations.

Thus, addressing these multifaceted challenges, from fundamental synthesis issues to complex in vivo behavior and regulatory complexities, is necessary for successful clinical translation and widespread adoption of nanoMIPs as a new frontier in brain disease therapy. Continuous research and collaborative efforts across various scientific and engineering disciplines are essential to navigate these hurdles.

## 8 Future Perspectives and Conclusion

The field of nanoMIPs for brain disease therapy is evolving; however, future directions will focus on integrating advanced functionalities, refining synthesis techniques, and personalized medicine approaches to fully unlock the potential of these “synthetic antibodies.” Numerous polymeric nanoparticles have already been developed and have successfully demonstrated their efficacy in drug delivery across the BBB, highlighting the immense potential of nanoMIPs as next‐generation targeted delivery systems. However, despite their promise, research and clinical translation efforts involving nanoMIPs remain relatively limited, highlighting the need for further investigation and optimization.

### 8.1 Integration with Multifunctional Theranostic Platforms

A significant future direction for MIP nanocarriers lies in their integration with multifunctional theranostic platforms. Theranostics combine therapeutic and diagnostic capabilities within a single system, allowing real‐time monitoring of drug delivery, therapeutic response, and disease progression.^[^ ^408^ ^]^ NanoMIPs are uniquely suited for this integration because of their tunable properties and specific recognition capabilities.

By incorporating imaging agents into nanoMIPs, researchers can achieve real‐time tracking of their biodistribution and accumulation within the brain. For instance, Lu et al. reported that fluorescent polymeric nanoparticles can be successfully applied as nanocarriers for transport across the BBB and have potential applications in brain imaging or drug delivery.^[^ ^227^ ^]^ Similarly, poly(butylcyanoacrylate) nanoparticles have been shown *in vivo* to facilitate the delivery of BBB‑impermeable contrast agents for optical imaging, including multiphoton fluorescence and whole‑brain MRI, thereby enabling molecular‐scale studies from individual synapses to the entire brain.^[^ ^409^ ^]^ When combined with the specific binding sites of MIPs, this allows for targeted imaging of diseased regions, enabling clinicians to confirm precise drug delivery and assess treatment efficacy noninvasively. Furthermore, these multifunctional platforms can incorporate components that respond to internal or external stimuli, enabling precise controlled drug release at the target site. This dual capability of diagnosis and therapy within one system offers a highly personalized and efficient approach to brain disease management, minimizing off‐target effects and maximizing therapeutic impact.

### 8.2 Innovations in Surface Engineering and Stimuli‐Responsive Systems

Future advancements will rely heavily on innovations in surface engineering and stimuli‐responsive systems to further optimize BBB permeation and control drug release. Surface engineering will continue to evolve beyond simple PEGylation and ligand conjugation. On the basis of the studies presented here, several novel strategies for future research approaches should be considered.

**Biomimetic coatings**. Designing nanoMIPs that mimic biological structures, e.g., cell membranes and viral capsids, could exploit natural cellular uptake pathways or evade immune surveillance more effectively. This could involve mimicking the surface properties of cells, such as neutrophils that naturally cross the BBB, allowing for “cellular hitchhiking”.

**Dynamic surface modifications**. The properties of developing surfaces, e.g., charge and hydrophobicity, can dynamically change in response to microenvironmental signals such as pH and enzyme activity to facilitate passage through different BBB layers or enhance cellular internalization.

**Peptide‐based targeting**. Further exploration of brain‐specific targeting peptides that can achieve high transcytosis efficiency without significant lysosomal degradation is needed, overcoming the current limitations of some antibody‐based approaches. Stimuli‐responsive systems will become more sophisticated, offering even better control over drug release.

**Multiple stimuli responsiveness**. Designing MIPs that respond to a combination of internal stimuli, e.g., pH and specific enzyme activity, or external triggers, e.g., focused ultrasound, light, magnetic fields, etc., can provide highly precise spatiotemporal control over drug release. Focused ultrasound (FUS) combined with microbubbles can transiently open the BBB locally and enhance nanoparticle distribution in the brain parenchyma.

**Feedback‐controlled release**. MIPs that can “sense” the concentration of a target molecule, e.g., a specific neurotransmitter or a tumor marker, can be developed, and their drug release rate can be adjusted accordingly, creating a truly intelligent, self‐regulated delivery system.

These innovations will contribute to the development of more efficient and safer nanoMIPs that can adapt to the complex and dynamic pathological microenvironments within the brain.

### 8.3 Advances in Real‐Time Imaging and Biosensing for Delivery Monitoring

Continued advances in real‐time imaging and biosensing are critical for the successful translation of nanoMIPs. While theranostic platforms integrate imaging, dedicated improvements in the imaging modalities themselves are necessary.

**Enhanced Resolution and Penetration** The development of imaging techniques with higher spatial and temporal resolution that are capable of penetrating deeper into brain tissue without invasiveness will allow for more precise tracking of nanocarriers at the cellular and even subcellular level.

**Quantitative imaging**. Moving beyond qualitative visualization to quantitative assessment of nanoMIP concentration, drug release, and target engagement in vivo is essential. The integration of nanoMIPs into biosensors is necessary for designing next‐generation smart healthcare facilities.

**Integration with Biosensors**. Pairing nanoMIPs with implantable or external biosensors for continuous, real‐time monitoring of biomarkers or drug concentrations in the brain microenvironment is needed. MIPs are extensively utilized in electrochemical and optical detection systems, demonstrating their potential for highly selective biosensing applications; therefore, such integration can provide immediate feedback on the efficacy of the delivered therapy and enable personalized dosage adjustments.

### 8.4 Personalized Medicine Approaches and Precision Neuropharmacology

The ultimate future direction for nanoMIPs is their role in personalized medicine approaches and precision neuropharmacology. The inherent specificity of nanoMIPs makes them uniquely suited for tailoring therapies to individual patient needs. In the long term, it might be possible to create nanoMIPs customized for an individual's unique disease profile, for example, by imprinting against patient‐specific biomarkers or even specific circulating tumor cells in the case of brain metastases. Furthermore, brain diseases often involve complex and heterogeneous pathologies. NanoMIPs can be designed to target multiple biomarkers simultaneously or to deliver different therapeutic agents via a combinatorial approach, addressing the multifaceted nature of these diseases. By precisely targeting diseased cells and controlling drug release, nanoMIPs can help overcome interpatient variability in drug response, leading to more predictable and effective treatment outcomes. The journey toward clinical realization for nanoMIPs is complex, requiring interdisciplinary collaboration between polymer chemists, neuroscientists, engineers, and clinicians. However, with continuous innovations in synthesis, functionalization, and monitoring technologies, MIP‐based nanocarriers are expected to significantly advance precision neuropharmacology and offer groundbreaking solutions for currently intractable brain diseases.

In conclusion, the treatment of brain diseases remains profoundly challenging due to the restrictive BBB, which blocks over 98% of small‐molecule drugs and nearly all macromolecular therapeutics from entering the CNS. Recent advances in nanoMIPs represent a transformative frontier in overcoming this limitation and redefining therapeutic strategies for neurological disorders, including neurodegenerative diseases, brain tumors, and CNS infections.

Since MIPs are synthetic receptors engineered to mimic the specificity and selectivity of natural biomolecules, which function via a “lock‐and‐key” mechanism, their molecular memory enables the creation of tailor‐made binding sites with high affinity for target molecules, ensuring precise drug delivery. In addition to being specific, nanoMIPs offer key advantages over conventional carriers: exceptional stability under harsh conditions, resistance to enzymatic degradation, reusability, and tunable nanoscale dimensions that facilitate efficient drug loading and rapid binding kinetics. Advances in fabrication techniques, particularly microfluidic‐based continuous synthesis, have improved scalability, reproducibility, and production efficiency, reducing synthesis times from hours to minutes.

To achieve effective BBB penetration, nanoMIPs employ multiple strategies:

- Surface modification with targeting ligands to exploit receptor‐mediated transcytosis via transferrin or insulin receptors.
- PEGylation prolongs circulation time by evading immune clearance.
- Cellular hitchhiking, where nanoMIPs associate with circulating neutrophils, enables active transport across the BBB to pathological sites.
- Stimuli‐responsive systems that release therapeutics in response to local microenvironmental cues, such as pH, enzymes, or redox conditions, enabling controlled, localized, and ‘on‐demand’ drug release.

The therapeutic potential of nanoMIPs spans a broad spectrum, including antivirals for CNS infections, chemotherapeutics for aggressive brain tumors, and neuroprotectants for degenerative conditions such as Alzheimer's disease and Parkinson's disease. Compared with conventional systemic therapies, their ability to concentrate drugs at diseased sites while minimizing systemic exposure improves therapeutic efficacy and reduces adverse effects.

Molecular imprinting offers a versatile platform for developing multifunctional theranostic systems that integrate diagnostic imaging and targeted therapy for personalized medicine. Continued progress in surface engineering and stimuli‐responsive designs will enable more efficient BBB crossing, selective tissue accumulation, and precise spatiotemporal drug release.

However, significant challenges remain before clinical translation:

- **Scalable and reproducible synthesis**: Developing standardized, cost‐effective, and reproducible manufacturing protocols is essential to ensure consistency in nanoMIP quality and performance.
- **Understanding protein corona dynamics**: Elucidating how protein corona formation alters nanocarrier behavior and reshapes drug release profiles remains a major hurdle, highlighting its role as an often overlooked determinant at the nanobiointerface.
- **Establishing long‐term safety profiles**: Comprehensive in vivo studies are needed to generate robust pharmacokinetic, biodistribution, and toxicity data to assess long‐term safety.
- **Navigating regulatory frameworks**: Overcoming regulatory barriers requires rigorous preclinical validation coupled with well‐structured clinical trial designs to demonstrate safety and efficacy.
- **Advancing imaging and tracking technologies**: Developing real‐time imaging and tracking strategies is crucial for confirming targeted brain delivery, monitoring biodistribution, and evaluating therapeutic outcomes.

Ultimately, nanoMIP‐based carriers hold the promise of precision neuropharmacology, delivering drugs exactly where and when they are needed while minimizing systemic side effects. For diseases once considered untreatable owing to BBB impermeability, nanoMIPs could unlock entirely new therapeutic avenues, improving patient quality of life and altering disease trajectories. The convergence of nanotechnology, polymer chemistry, neurobiology, and clinical medicine will drive this innovation forward, firmly establishing nanoMIPs as a next‐generation platform for targeted drug delivery and personalized CNS therapies.

## Conflict of interest

The authors declare no conflict of interest.

## Author Contributions

R.D. conceived and prepared the manuscript, prepared figures, and summarized the tables. R.D., S.S., and K.‐T.K. discussed the concepts and edited the final version. R.D. and K.‐T.K. supervised, edited, and all the authors have approved the version for submission.
